# Genome and transcriptome-based characterization of high energy carbon-ion beam irradiation induced delayed flower senescence mutant in *Lotus japonicus*

**DOI:** 10.1186/s12870-021-03283-0

**Published:** 2021-11-03

**Authors:** Yan Du, Shanwei Luo, Jian Zhao, Zhuo Feng, Xia Chen, Weibin Ren, Xiao Liu, Zhuanzi Wang, Lixia Yu, Wenjian Li, Ying Qu, Jie Liu, Libin Zhou

**Affiliations:** 1 Biophysics Group, Biomedical Center, Institute of Modern Physics, Chinese Academy of Sciences, 730000 Lanzhou, People’s Republic of China; 2 University of Chinese Academy of Sciences, Beijing, 100000 People’s Republic of China; 3 Guangdong Key Laboratory for New Technology Research of Vegetables, Vegetable Research Institute, Guangdong Academy of Agricultural Sciences, Guangzhou, 510640 Guangdong China; 4 School of Life Science and Engineering, Lanzhou University of Technology, Lanzhou, 730000 People’s Republic of China; 5 Kejin Innovation Institute of Heavy Ion Beam Biological Industry, Baiyin, 730900 People’s Republic of China; 6 Institute of Geographic Sciences and Natural Resources Research, Chinese Academy of Sciences, Beijing, 100000 People’s Republic of China

**Keywords:** *Lotus japonicus*, Flower senescence-delayed, Carbon-ion beam irradiation, Whole genome re-sequencing, RNA-seq, Phytohormone

## Abstract

**Background:**

Flower longevity is closely related to pollen dispersal and reproductive success in all plants, as well as the commercial value of ornamental plants. Mutants that display variation in flower longevity are useful tools for understanding the mechanisms underlying this trait. Heavy-ion beam irradiation has great potential to improve flower shapes and colors; however, few studies are available on the mutation of flower senescence in leguminous plants.

**Results:**

A mutant (*C416*) exhibiting blossom duration eight times longer than that of the wild type (WT) was isolated in *Lotus japonicus* derived from carbon ion beam irradiation. Genetic assays supported that the delayed flower senescence of *C416* was a dominant trait controlled by a single gene, which was located between 4,616,611 Mb and 5,331,876 Mb on chromosome III. By using a sorting strategy of multi-sample parallel genome sequencing, candidate genes were narrowed to the gene CUFF.40834, which exhibited high identity to ethylene receptor 1 in other model plants. A physiological assay demonstrated that *C416* was insensitive to ethylene precursor. Furthermore, the dynamic changes of phytohormone regulatory network in petals at different developmental stages was compared by using RNA-seq. In brief, the ethylene, jasmonic acid (JA), and salicylic acid (SA) signaling pathways were negatively regulated in *C416*, whereas the brassinosteroid (BR) and cytokinin signaling pathways were positively regulated, and auxin exhibited dual effects on flower senescence in *Lotus japonicus*. The abscisic acid (ABA) signaling pathway is positively regulated in *C416*.

**Conclusion:**

So far, *C416* might be the first reported mutant carrying a mutation in an endogenous ethylene-related gene in *Lotus japonicus*, rather than through the introduction of exogenous genes by transgenic techniques. A schematic of the flower senescence of *Lotus japonicus* from the perspective of the phytohormone regulatory network was provided based on transcriptome profiling of petals at different developmental stages. This study is informative for elucidating the molecular mechanism of delayed flower senescence in *C416*, and lays a foundation for candidate flower senescence gene identification in *Lotus japonicus*. It also provides another perspective for the improvement of flower longevity in legume plants by heavy-ion beam.

**Supplementary Information:**

The online version contains supplementary material available at 10.1186/s12870-021-03283-0.

## Background

Flower longevity is the duration of time that flowers remain open and functional. It is an important trait for ornamental plants because of its effects on floral displays. Extending flower longevity will greatly enhance the commercial value and competitiveness of ornamental plants. Furthermore, flower longevity contributes significantly to pollen dispersal and reproductive success in flowering plants. Flower senescence is a highly programmed process that is precisely controlled. Developmental (reproduction, developmental cues, growth regulators, etc.) and environmental (pollination, drought, mechanical injury, etc.) stimuli function as signals to initiate the breakdown and remobilization of cellular constituents, causing serial changes in morphological, cytological, physiological, and molecular aspects, including hormonal level imbalances; loss of membrane permeability; higher levels of oxidative and reduced levels of protective enzymes; loss of nucleic acids, proteins, and organelles; visible cell death symptoms [1]. Plant hormones, especially ethylene, are the most crucial regulators of flower senescence [2, 3]. Patterns of flower senescence are categorized as ethylene-sensitive and ethylene-insensitive [4]. For ethylene-sensitive plant species, the increase in endogenous ethylene promotes flower senescence, and chemical or genetic modification of ethylene biosynthesis or perception genes can alter the process of flower senescence [1, 4, 5]. In petunias, the transcription factor PhFBH4 interacts with the G-box cis-element in the promoter of 1-Aminocyclopropane 1-carboxylic acid (ACC) synthase 1, which is a key factor in ethylene biosynthesis. The silencing of *PhFBH4* extends flower longevity, whereas overexpression lines exhibit a shorter flower lifespan [6]. Ethylene-sensitive rose cultivars showed changes in the expression of *RhACO1* (1-aminocyclopropane-1-carboxylate oxidase 1) [7]. Ethylene is perceived by receptors and then acts via CTR1 (constitutive triple response 1), which is a negative regulator of ethylene signaling. Downstream of the receptor-CTR1 complex is EIN2 (ethylene insensitive 2), which is a positive regulator of the ethylene response. Finally, the ethylene response is mediated by a series of transcriptional factors, such as EIN3 (ethylene insensitive 3) and ERF1 (ethylene response factor 1) [8, 9]. Genetic manipulation of ethylene signaling transduction factors can also modulate petal senescence [10–12]. Low levels of ethylene production are a common feature of ethylene-insensitive plants, and flower longevity usually does not respond to the application of ethylene inhibitors or exogenous ethylene. It has been reported that, cysteine protease has the potential to regulate ethylene biosynthesis and signal transduction by acting as a dual-function protein with enzymatic activity and transcriptional factor activity that bonded to the cis element to activate the expression of ACC synthesize gene. Meanwhile, the expression of cysteine proteinase is also regulated by ethylene and associated with programmed cell death or plant senescence in *Arabidopsis*, *Dianthus caryophyllus* L., *Cicer arietinum* L., and *Brassica napus* [13–17]. In addition, ethylene had been demonstrated to play special roles in regulating symbiotic nitrogen-fixation and nodulation organogenesis in legumes while influencing flower senescence [18–20]. Instead, abscisic acid (ABA) may play a primary role in these species [21]. To date, most of the knowledge of flower senescence is based on limited plant species. However, plants always make a tradeoff between investing resources in developing and maintaining flower function, and flower longevity is susceptible to climatic factors such as temperature and water availability; therefore, flower longevity is always limited in a specie specific manner for energetic and ecological reasons in nature [22, 23]. Few studies are available on flower senescence of leguminous plants, which cover 20,000 species and play important roles in agronomic activities and environmental improvement due to their nitrogen-fixing ability and ornamental value. *Lotus japonicus* is a valuable legume that has been used as pasture for over 30 years [24]. Moreover, *Lotus japonicus* is also used as an ornamental plant for decorating houses and cities due to its abundant bright yellow flowers. To date, flower longevity in *Lotus japonicus* has not been fully characterized. Stable mutants displaying alterations in flower longevity are ideal tools for understanding the mechanism of flower longevity in *Lotus japonicus*.

Unrestricted to species that have a mature genetic transformation system and high-quality genome information, heavy-ion beam irradiation can induce mutations in diverse plant species by multiform sample status. Heavy-ion beam consist of ionized particles produced by heavy-ion accelerators, such as cyclotrons and synchrotrons. Because the amount of energy deposited at a unit distance of heavy-ion beam is much more intensive than that of γ-rays and X-rays with low linear energy transfer (LET), heavy-ion beam irradiation can induce severe DNA damage, including DNA double-stranded breaks (DSBs) and clustered damage. Therefore, heavy-ion beam has a higher relative biological effectiveness (RBE) than γ-rays and X-rays [25]. If this damage is inaccurately repaired or unrepaired, mutations such as single-base substitutions (SBSs), insertions and deletions, and rearrangements are commonly induced in the genome. Some of these mutations can result in alterations in phenotype, traits and resistances. In the past decades, many mutation resources have been generated through heavy-ion beam irradiation in plants [26–37]. In our previous study, we constructed an M_2_ seed collection of *Lotus japonicus* by carbon-ion beam irradiation produced by the Heavy Ion Research Facility in Lanzhou (HIRFL). A flower senescence-delayed mutant line, *C416*, was isolated from the M_2_ population [35]. It is an ideal tool to study the mechanism of heavy-ion beam irradiation with respect to flower longevity and to provide a better understanding of the process and mechanism of senescence in *Lotus japonicus*.

The identification of casual genes is still a great challenge for non-model plant species and those with low-quality reference genome information. In recent years, next- generation sequencing (NGS) techniques have enabled highly reliable analytical measurements of genome variants, as well as the transcriptome in massively parallel sequencing. It has become an effective and economical tool for exploring the regulatory associations between genomic features, gene expression patterns, and phenotypic traits. For instance, by the combined use of whole-genome re-sequencing and transcriptome analysis, genes related to cadmium tolerance in *C. reinhardtii* and resistance to soybean (*Glycine max*) mosaic virus strain SC3 [38, 39], a novel single-base mutation induced by EMS in *CaBRI1* that resulted in brassinosteroid accumulation, was responsible for the dwarf phenotype in peppers [40]. To explore the causal gene for the phenotype of *C416*, genetic mapping was carried out by association analysis of multi-sample parallel genome sequencing and rough mapping. The candidate genes were narrowed to a gene exhibited high identity to *ETR1* in *Glycine max*, *Medicago truncatula* and *Arabidopsis thaliana*. A physiological assay was performed to test the sensitivity to ethylene precursors. Furthermore, transcriptome profiling by RNA sequencing was performed to understand flower senescence in *Lotus japonicus* from the perspective of the phytohormone regulatory network. These results are informative for elucidation of the molecular mechanism of delayed flower senescence in *C416* and lay a foundation for candidate flower senescence gene identification in *Lotus japonicus*.

## Results

### Phenotypic characterization of the *C416* mutant

**Fig. 1:**
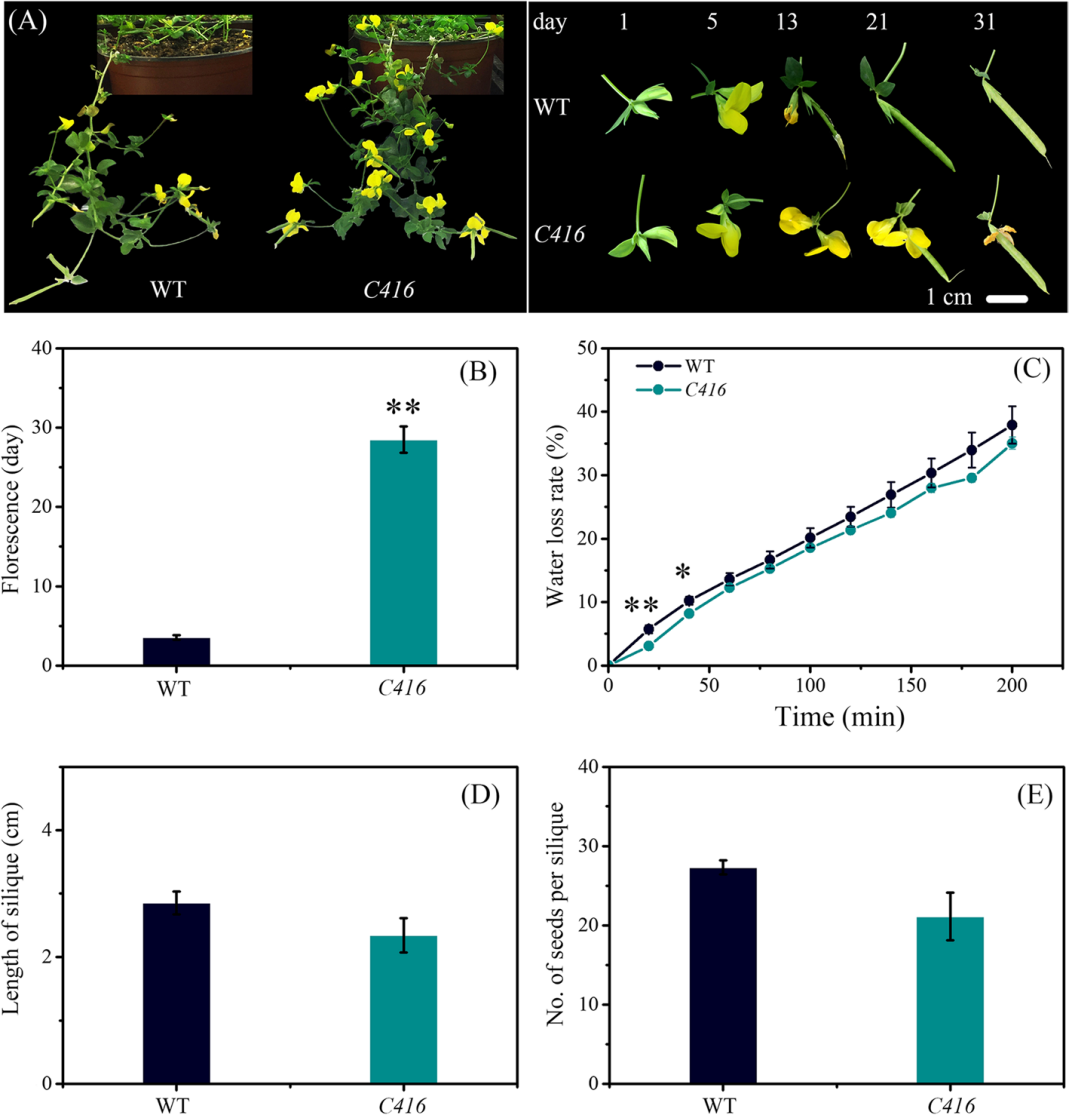
Phenotypic and physiological characteristics of reproductive stages. **A** Phenotypes of WT and *C416*. **B** Floral longevity of WT and *C416*. **C** Water loss rate of detached flowers of WT and *C416*. **D** Pod length, **E** Average number of seeds per pod. The data points are mean ± standard error of three replications

The *C416* mutant was induced by carbon-ion beam irradiation in our previous study. Compared to the WT, *C416* displayed distinct delayed flower senescence, with an average blossom duration of approximately 8.03 folds of the WT. Flowers of *C416* did not exhibit abscission even until the plants died (Fig. 1A, B). The water loss rate of detached flowers did not differ between *C416* and WT, as well as the size of pods and the number of seeds per silique (Fig. 1C-E). Interestingly, during the silique development process, curved pods were always observed in *C416*, although these pods would eventually straighten. These phenotypic characteristics of *C416* could be stably inherited by its progeny, even after continuous selfing for six generations.

### Genetic analysis and rough mapping of *C416*

**Fig. 2:**
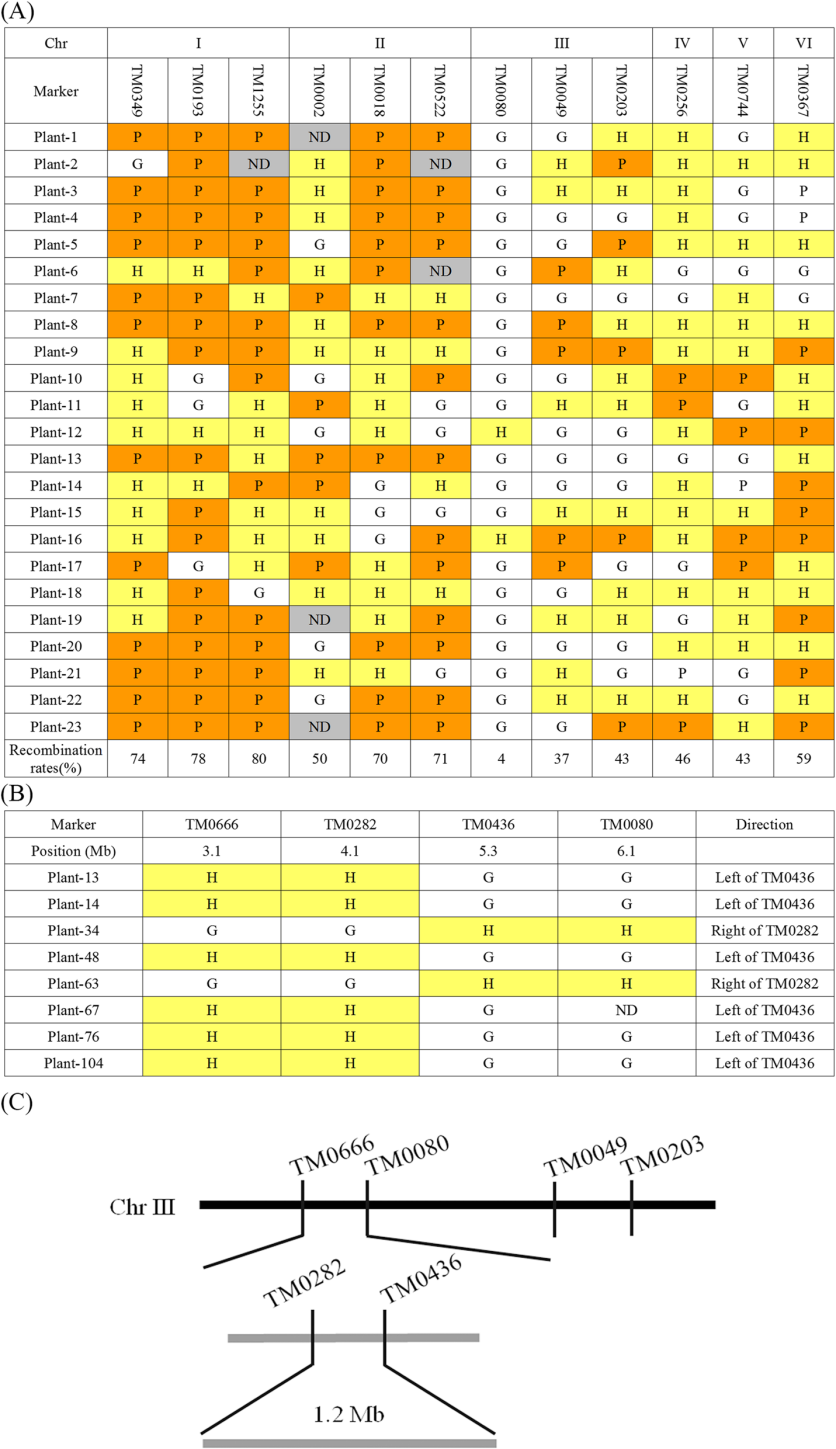
Genetic mapping of *C416*. **A** Colormap of the initial mapping of the *C416* mutation. **B** Colormap of genotyping based on amplified bands by markers in chromosome III. From top down are the chromosome number, TM marker number, and number of individual plant tested with microsatellite markers from different chromosomal regions. P: homozygous *Lotus japonicus* MG-20, G: homozygous *Lotus japonicus* Gifu B-129, H: heterozygous, ND: not detected. $\mathrm{Recombination}\;\mathrm{rate}\;=\;\frac{\left(\mathrm H+2\mathrm P\right)}{2\mathrm N}$, ‘N’ indicates the total number of plants. **C** Overview of genetic mapping of *C416*

The first steps for exploring the causal gene of *C416* included genetic analysis and rough mapping. It displayed a stable mutant phenotype even after continuous selfing for six generations. *C416* was back-crossed with WT MG20, followed by a reciprocal cross with a polymorphic mapping parent, Gifu. Based on delayed flower senescence, F_1_ and F_2_ plants were genotyped unambiguously. All F_1_ plants showed a mutant phenotype, indicating that the mutation of *C416* was dominant. A total of 279 F_2_ individual plants were cultured, and the genetic segregation ratios of the mutant phenotype to those of the WT were 3:1 (Table 1). These results indicated that the mutant trait of *C416* was under the control of a single dominant gene. F_2_ plants with the WT phenotype were selected for rough mapping because they were homozygous for the recessive allele. First, by using SSR markers and 23 individual plants, the mutation locus was roughly positioned on chromosome III and closely linked to the marker TM0080 based on the lowest exchange rate (4%) (Fig. 2A and Fig. S1). To further narrow down the region that contains the responsible gene, amplified mapping population and dense markers were used to find the closer recombination end-points on either side of the target gene (*C416*) (Fig. 2B). Finally, the candidate region was narrowed down to between TM0282 and TM0436, with an interval of 1.2 centimorgan (cM) (Fig. 2C).

**Table 1:** The number of different phenotypic seedlings segregated in the offspring of a reciprocal cross

Cross	F_1_ phenotype	Number of seedlings in F_2_		Expect ratio of *C416*/WT	Chi-square (*P*-value)
		WT	*C416*		
*C416*(♂)xMG20(♀)	*C416*	15	34	3:1	0.823 (*P* = 0.364)
MG20(♂)x*C416*(♀)	*C416*	10	31	3:1	0.008 (*P* = 0.928)
Gifu(♀)x*C416*(♂)	*C416*	51	138	3:1	0.397 (*P* = 0.529)

### Association analysis of genetic mapping and re-sequencing of *C416*

**Fig. 3:**
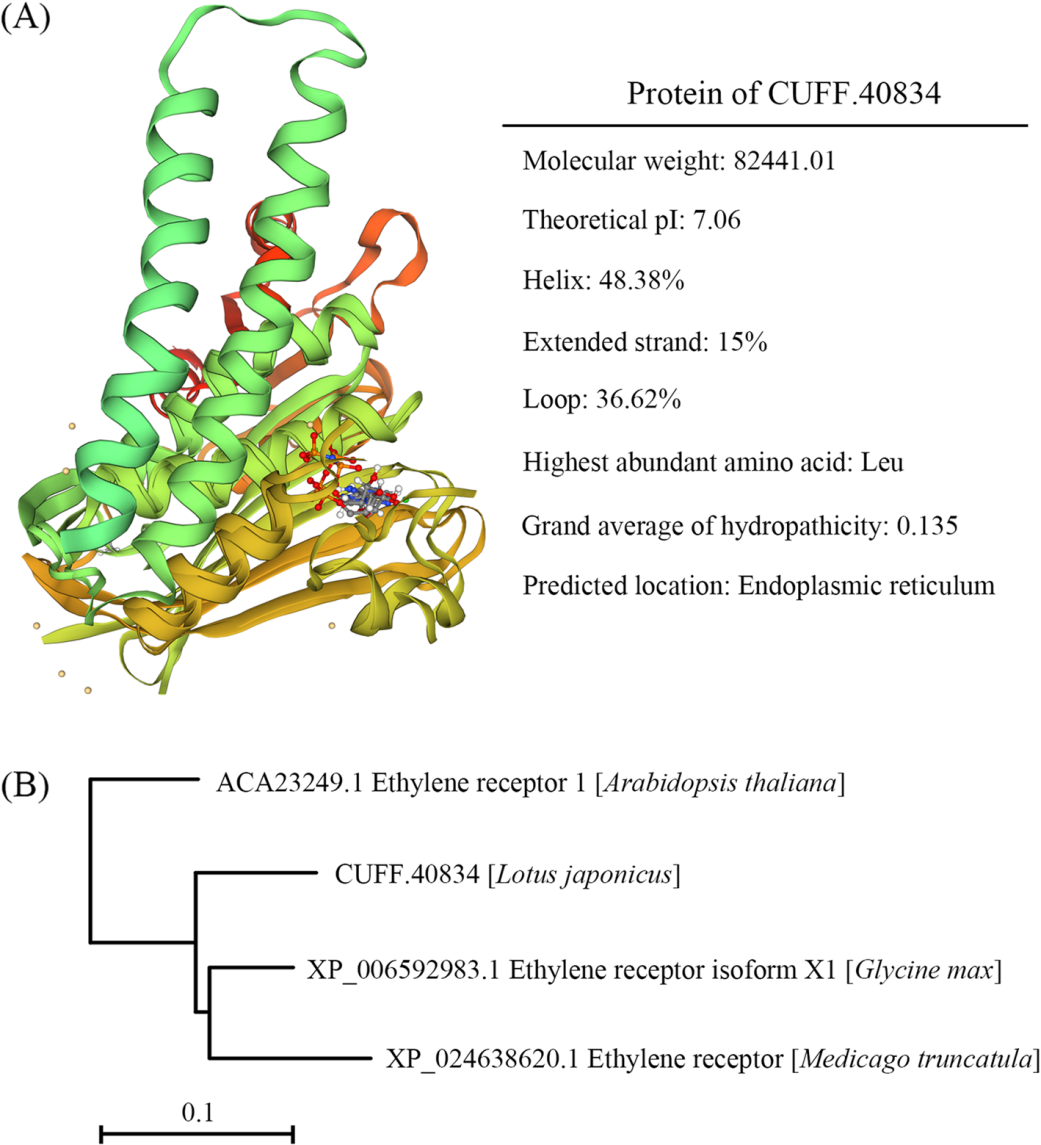
Bioinformatic analysis of CUFF.40834 protein. **A** Structure prediction, subcellular localization prediction and physicochemical property analysis of CUFF.40834. **B** Phylogenetic analysis based on amino acid alignment, unrooted phylogenetic tree of CUFF.40834 and its homologous proteins in *Arabidopsis thaliana*, *Glycine max* and *Medicago truncatula*

We re-sequenced *C416*, as well as five other lines derived from carbon-ion beam irradiation. Mutations that were unique to *C416* were detected by ruling out the background mutations shared by any two samples, as reported in our previous studies [41]. Several variants were randomly selected for verification by Sanger sequencing (Fig. S2) to verify the reliability of the re-sequencing. As *C416* is a stable mutant line, only homozygous variants in the genome were considered in the present study. A total of 261 homozygous mutations composed of 191 SBSs (73.18%), 55 deletions (21.07%), and 15 insertions (5.75%) were detected in six plants. Among the 261 homozygous mutations, 62 variants consisting of 49 SBSs, 10 deletions, and 3 insertions, were unique to the *C416* genome. In detail, there were 21, 16, 6, 6, 8, 2, and 3 mutations located in chromosome 0, I, II, III, IV, V and VI, respectively (Table S1). Fifty-six candidate genes remained after ruling out those located in the intergenic and intron regions. Associated with the rough mapping of *C416*, we focused on the mutated genes on chromosome III and as well as chromosome 0, as the *Lotus japonicus* genome assembly version 3.0 has 162 Mb of the genome assigned to a virtual chromosome 0. Finally, only 22 genes were sorted out. Among these, only 12 genes had functional annotations or predictions (Table 2). In the rough mapping region, CUFF.40834 showed a thymine → cytosine single base substitution in exon resulting in a change in amino acid from leucine to serine. The structure and physicochemical property prediction of CUFF.40834 protein was shown in Fig. 3A. Bioinformatics analysis showed that the theoretical molecular weight of CUFF.40834 protein was 824,441.01, theoretical isoelectric point was 7.06, the highest abundant amino acid was Leu, accounting for 12.4%, the grand average of hydropathicity was 0.135. In addition, CUFF.40834 was predicted to subcellular localized on endoplasmic reticulum. The protein blast results showed that CUFF.40834 exhibited high homology to ETR1, which was well characterized in *Arabidopsis thaliana*, *Glycine max* and *Medicago truncatula* (Fig. 3B). This mutation was indirectly verified by RT-qPCR by designing primers around the mutation site (Fig. S3). By combining of the above results, we preliminarily inferred that CUFF.40834 might be the causal gene for the extended flower longevity of *C416*.

**Table 2:** Homozygous mutations located in chromosome 0 and chromosome III detected in *C416* genome by re-sequencing^a^

Chr	Position	Reference	Alteration	Type	ID	Function annotation
0	5,211,768	T	A	SBS	CUFF.738	Zinc finger CCCH domain-containing protein
0	18,170,144	A	T	SBS	CUFF.2453	Cytochrome P450 94A1-like
0	48,986,237	G	A	SBS	CUFF.6544	Chloroplast-targeted copper chaperone protein
0	116,649,426	T	C	SBS	CUFF.14705	TashAT2
0	125,393,533	G	A	SBS	CUFF.15787	F-box
0	146,760,633	G	A	SBS	CUFF.18858	F-box/FBD/LRR-repeat protein At5g56420-like
0	149,463,384	G	-15^b^	Deletion	CUFF.19232	Cysteine proteinase precursor
0	157,428,073	A	T	SBS	CUFF.20352	Probable protein phosphatase 2C 60-like isoform X1
0	174,990,604	T	C	SBS	CUFF.22836	EMB1273
3	4,271,744	T	G	SBS	CUFF.40793	TMV resistance protein N-like
3	38,052,958	T	+TTC	Insertion	CUFF.45186	DNA ligase
3	4,982,946	T	C	SBS	CUFF.40834	Signal transduction response regulator

^a^Only mutations that located in genes with definite function prediction were shown here

^b^Insertions or deletions in size above 5 bp were displayed by ‘+’ or ‘-‘followed by the length of mutation fragments

### Sensitivity of *C416* and WT to the ethylene precursor

**Fig. 4:**
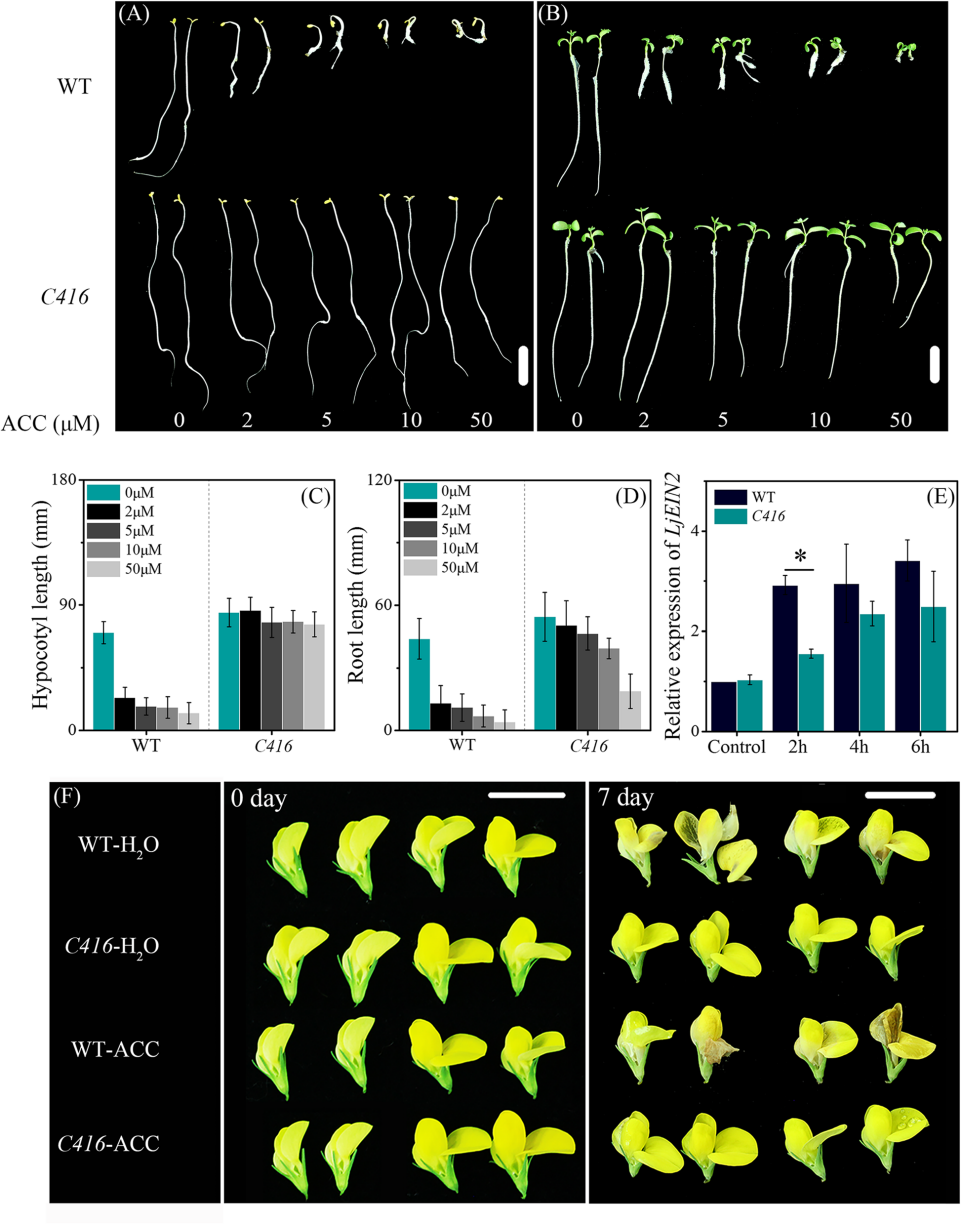
Sensitivity of WT and *C416* to ACC. Phenotype of hypocotyls (**A**) and root (**B**). **C** Hypocotyls length of seedlings germinated on ACC with different concentration for 10 days (in the dark). **D** Root length of seedlings germinated on ACC with different concentration for 10 days (under light condition) (**E**) Relative expression level of *LjEIN2*. Seedlings were transferred to the 1/2 MS medium with 0 and 5 μM ACC and the expression level of *LjEIN2* was examined at the indicated time. Control represents untreated seedlings. Data are means ± standard error of three biological replications. **F**, Response of detached flower in different stages to ACC treatment. Scale bar, 1 cm

Based on the indication provided by the genetic mapping, we examined the sensitivity of both *C416* and WT to the ethylene precursor ACC. Triple response (hooks, lateral expansion, and dwarfing) assay was carried out in the early developmental stage. The *C416* and WT were germinated on 1/2 MS containing 0—50 μM ACC in the dark for 10 days. The dark-grown seedlings of *C416* did not respond to the application of ACC, as no difference was observed between the treatment and control groups. The WT showed hooked, thickened, and dwarf hypocotyls after treatment with ACC (Fig. 4A, C). In addition, in light conditions, *C416* also showed a significant difference in response to ACC in root development, including the length and root hair, when compared with the WT (Fig. 4B, D). The expression level of *EIN2*, an important gene involved in ethylene biosynthesis and signal transduction, was also examined (Fig. 4E). The extent of the WT response to ACC appeared stronger than that of the *C416* based on the expression patterns of *LjEIN2* (CUFF.32775); the expression was significantly lower in the *C416* after 2 h of treatment with 5 μM ACC than that in the WT. During the reproduction stage, the detached flowers were treated with 100 μM ACC and ddH_2_O for 1 week. ACC treatment did not promote senescence of the detached flowers in *C416*, as no brown area was observed in the petals, whereas obvious brown area appeared in the WT petals in the ACC treatment group (Fig. 4F). These results indicate that *C416* is insensitive to ethylene, which may be caused by abnormal ethylene signal transduction.

### Comparison of petal transcriptional profile between WT and *C416*

**Fig. 5:**
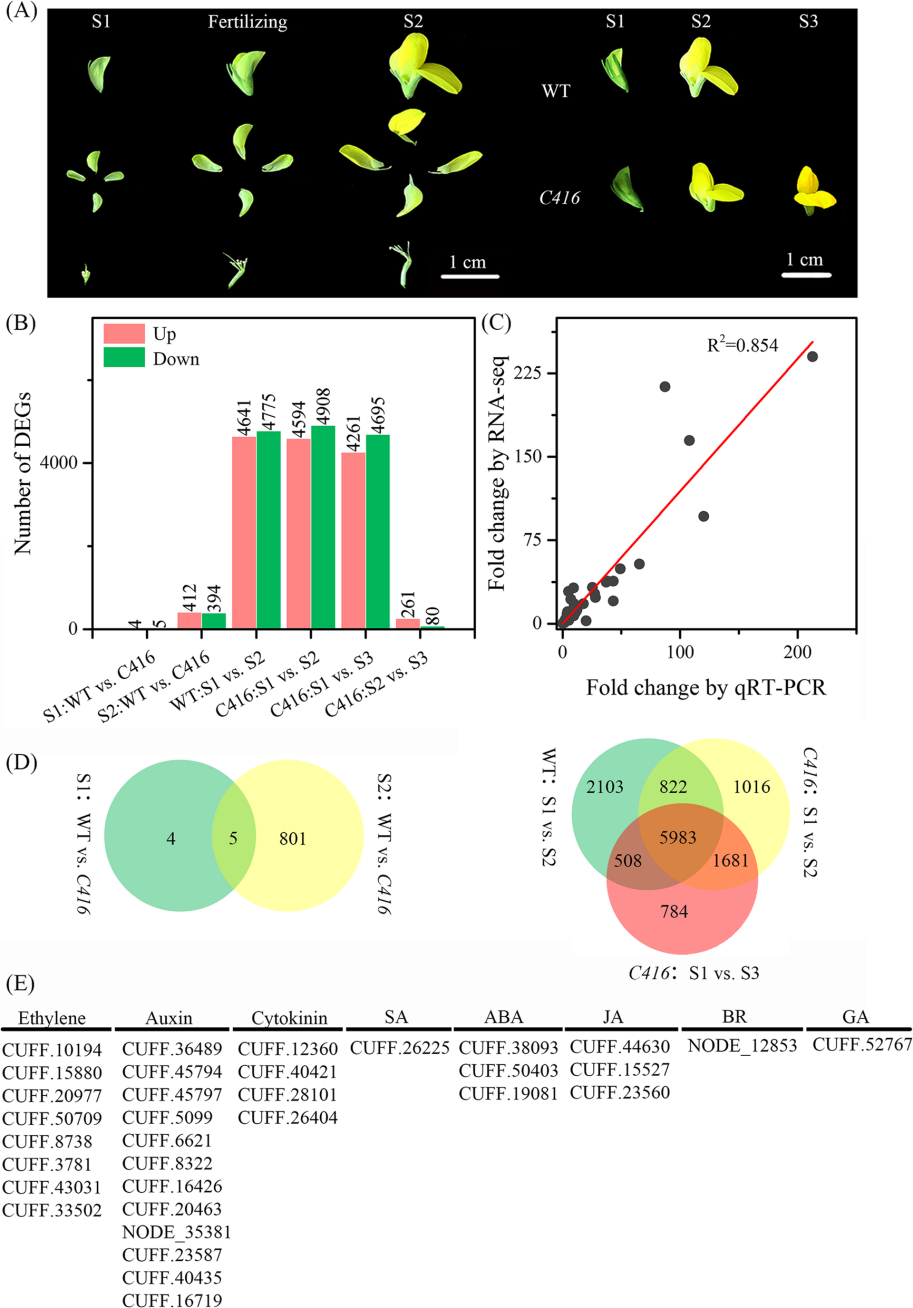
A global view of transcriptome dynamics in petals of WT and *C416*. **A** Stage of flower development in *Lotus japonicus* and samples used for RNA-Seq. **B** Statistical analysis of DEGs at different stages. **C** The correlation coefficient between RNA-seq and RT-qPCR data base on 18 genes. **D** Venn diagrams showing the distribution of DEGs in WT-S1 vs. *C416*-S1 and WT-S2 vs. *C416*-S2 (left), and in WT-S1 vs. WT-S2, *C416*-S1 vs. *C416*-S2 and *C416*-S1 vs. *C416*-S3 (right). **E** List of DEGs involved in phytohormone signal transduction

To explore the molecular aspects of the *C416* petal senescence process based on the phytohormone regulation, transcriptome profiles were generated by RNA-seq analysis. Petals were harvested 2 days before pollination (S1), 2 days after pollination (S2) and 8 days after pollination (S3, only for *C416*) were used for RNA-Seq (Fig. 5A). A total of 917,203,062 raw reads were generated for 15 petal samples and 22 to 44 million clean reads for each sample (Supplementary Table A.1). The numbers of differentially expressed genes (DEGs) were shown in Fig. 5B. The biggest difference was detected in S1 vs. S2 and S1 vs. S3. For instance, in *C416*-S1 vs. *C416*-S2, there were 9502 DEGs were detected, whereas, only 341 DEGs were detected in *C416*-S2 vs. *C416*-S3 (Fig. 5B). In addition, for WT vs. *C416*, there were more DEGs were detected in S2 than that in S1. In detail, four and five genes exhibited up- and down-regulated expression patterns at S1, respectively, whereas, 412 and 394 DEGs were up- and down-regulated at S2 (Fig. 5B). Eighteen genes were selected to test the correlation coefficient between RNA-seq and RT-qPCR, the R^2^ was calculated as 0.85, and expression patterns showed high consistency, all of which indicated that the results of RNA-seq are reliable for further study (Fig. 5C, Fig. S4 and Fig. S5). In addition, five DEGs were shared between these two stages (Fig. 5D). In S1 vs. S2, more than 9000 DEGs were detected in both the WT and *C416*, and 6805 DEGs were shared by these two lines (Fig. 5D).

### Flower senescence in *Lotus japonicus* from the perspective of plant hormone regulatory network

**Fig. 6:**
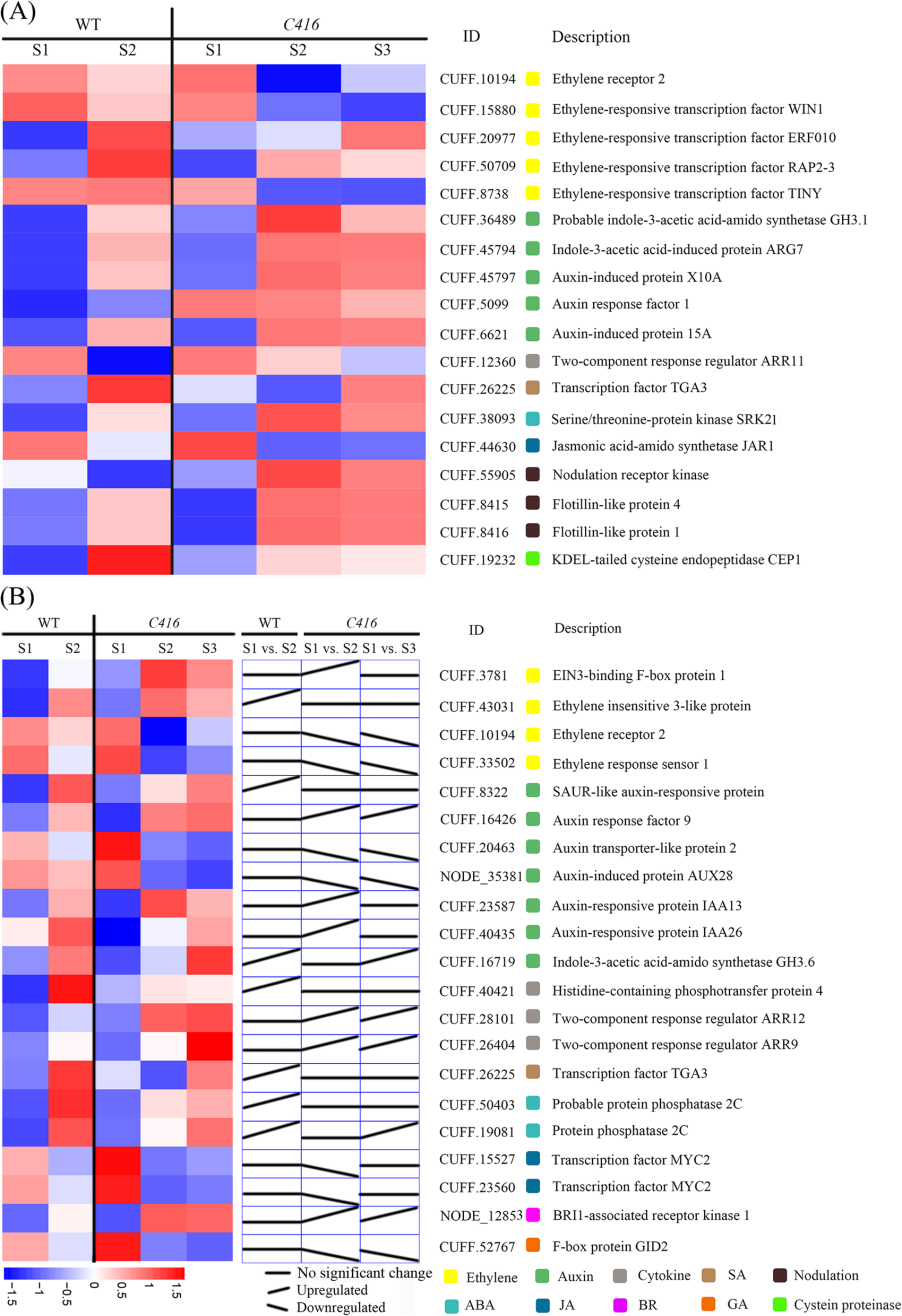
Expression pattern of phytohormone-related genes during petal senescence. **A**, Expression level of DEGs in both of WT-S1 vs. *C416*-S1 and WT-S2 vs. *C416*-S2. **B**, Expression analysis of genes with different expression trend between WT-S1 vs. WT-S2 and *C416*-S1 vs. *C416*-S2

**Fig. 7:**
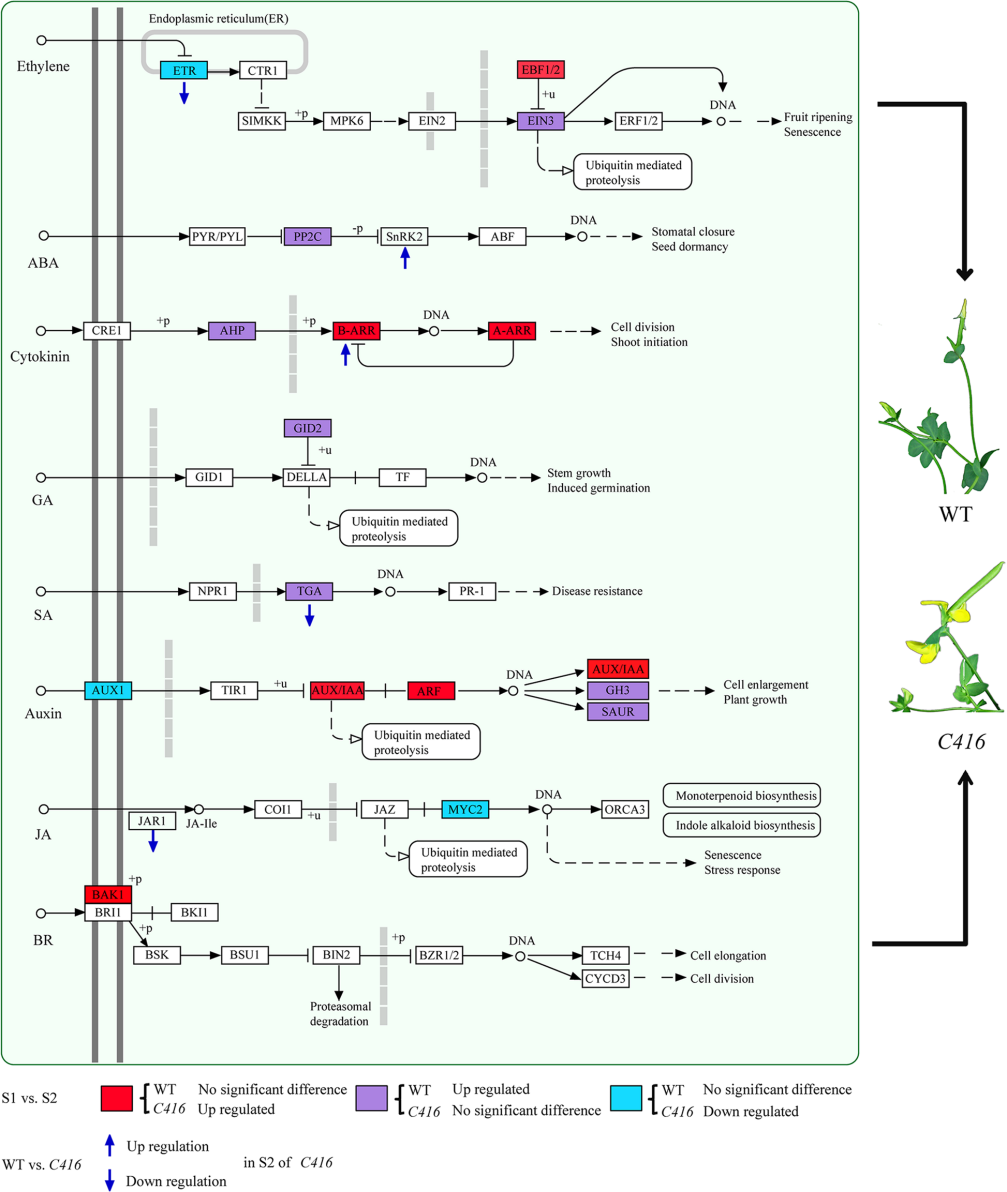
Schematic of expression profile of plant hormone signaling transduction genes in flower senescence delayed mutant *C416*. Circles represent metabolites, and box represent regulatory genes

DEGs in WT-S1 vs. *C416*-S1 and WT-S2 vs. *C416*-S2, as well as those DEGs with different expression models among WT-S1 vs. WT-S2, *C416*-S1 vs. *C416*-S2 were mainly considered in the present study. Among these, DEGs involved in phytohormone signal transduction processes were screened out in the subsequent analysis, as they play important roles in plant senescence (Fig. 5E). In the case of WT-S1 vs. *C416*-S1 and WT-S2 vs. *C416*-S2, five DEGs were identified in the ethylene-mediated signaling pathway; five in the auxin-mediated signaling pathway; and one in salicylic acid (SA), jasmonic acid (JA), cytokinin, and ABA -mediated signaling pathways (Fig. 6A). Figure 6A shows the expression patterns of all these genes, as well as genes involved in nodulation, cysteine proteinase as they showed definite regulatory effects on ethylene (biosynthesis or signaling transduction) and flower senescence in previous studies. Ethylene receptor *ETR2*, ethylene-responsive transcription factors *WIN1*, *TINY*, *RAP2–3*, and *ERF010* were downregulated in *C416*-S2 when compared with those of WT-S2. CUFF.19232, which encodes a cysteine protease, was down regulated in *C416*-S2. CUFF.8415 and CUFF.8416 which involved in nodulation were up regulated in *C416*-S2. *TGA3* (SA response factor) and *JAR1* (jasmonic acid amino synthetase) were also downregulated in *C416*-S2. Auxin response factor *ARF1*, auxin-induced protein *15A* and *X10A*, *ARG7* and *GH3.1*, two-component response regulator *ARR11* (cytokinin signaling pathway), and SNF1-related protein kinase *SRK21* (ABA signaling pathway) were upregulated in *C416*-S2. In the case of WT-S1 vs. WT-S2 and *C416*-S1 vs. *C416*-S2 (Fig. 6B), four DEGs were involved in the ethylene signaling pathway (*EBF1*, *EIN3*, *ETR2*, and *ERS1*), seven in the auxin signaling pathway (*SAUR-like* gene, *auxin transporter-like protein 2*, *AUX28*, *IAA13*, *IAA26*, and *GH3.6*), three in cytokinin signaling pathway (*AHP4*, *ARR9*, and *ARR12*), one in the SA signaling pathway (*TGA3*), two in the ABA signaling pathway (Protein phosphatase 2C, *PP2C*), two in the JA signaling pathway (*MYC2*), one in the brassinosteroids (BR) signaling pathway (*BAK1*) and one in the gibberellic acid (GA) signaling pathway (*GID2*) exhibited different expression change tendencies between the WT and *C416* (Fig. 6B). Overall, the *C416* mutation led to differential expression of a series of genes that are involved in multiple phytohormone signaling pathways (Fig. 7). In brief, ethylene, JA, and SA signaling pathways were negatively regulated in *C416*, whereas the BR and cytokinin signaling pathway were positively regulated, and auxin exhibited dual effects on flower senescence in *Lotus japonicus*. The ABA signaling pathway was positively regulated in *C416*, and ABA might be the major phytohormone factor that promotes senescence when the ethylene signaling pathway is blocked in *Lotus japonicus*.

## Discussion

Petal senescence is the last stage of flower development and is a precisely regulated and irreversible programmed process. The life span of flowers is limited by species-specific, ecological and energetic factors [42]. Mutants displaying longer or shorter flower life expectancy are useful tools for understanding the mechanism of the senescence process. It also greatly improves the commercial value of ornamental plants. Compared with those for flower shape and color, mutations in flower senescence have seldom been reported in the history of mutation breeding by heavy-ion beam irradiation. By using carbon-ion beam irradiation, a mutant (*C416*) that exhibited delayed flower senescence and abscission was isolated in *Lotus japonicus*; its florescence extended to almost eight times longer than that of the WT.

Precise exploration of the causal gene through omics studies might be relatively more difficult for *Lotus japonicus* as 41.12% of the genome was unanchored to a specific chromosome. The *Lotus japonicus* genome assembly version 3.0 was constructed by a hybrid assembly integrating Sanger-sequencing data from TAC/BAC clones and shotgun approaches with Illumina sequencing data to 40 × genome coverage. A total of 132 scaffolds covering 232 Mb were aligned to six *Lotus japonicus* chromosomes. The 23,572 contigs, corresponding to 162 Mb of the genome, were assigned to the virtual chromosome 0 [43]. In this study, we integrated and associated the results in the following aspects: Genetic assay supported that the mutation phenotype of *C416* was a dominant trait controlled by a single gene, which was located between 4,616,611 Mb and 5,331,876 Mb in chromosome III (Fig. 2 and Table 1). Using a sorting strategy of multi-sample parallel genome sequencing reported in our previous study, 61 homozygous variants were detected in *C416*; only the mutations located in chromosome III and 0 were considered for further screening (Table 2). CUFF.40834 showed a thymine → cytosine single base substitution in the exon, resulting in a change in amino acid from leucine to serine and was regarded as the candidate gene. BLAST analysis of the CUFF.40834 amino acid sequence on the NCBI website revealed that it exhibited high identity to ETR1 in *Glycine max*, *Medicago truncatula* and *Arabidopsis thaliana* (Fig. 3B), and physiological assays demonstrated that *C416* was insensitive to ACC (Fig. 4). Transcriptome profiling of petals at different developmental stages in the WT and *C416* revealed that ethylene signaling transduction was inhibited in *C416*, which might be responsible for the delayed flower senescence in *C416* (Fig. 6). In *Arabidopsis thaliana*, there are five membrane-bound ethylene receptor isoforms, including ETR1, ERS1 (ethylene response sensor 1), EIN4 (ethylene insensitive 4), ETR2, and ERS2, which regulate the activity of the downstream mitogen-activated protein kinase kinase kinase, CTR1. They function as negative regulators of ethylene signaling [9]. Dominant mutations in *ETR1*, *ETR2*, and *EIN4* caused ethylene insensitivity in *Arabidopsis thaliana*. Among these receptors, ETR1 plays a predominant role in ethylene perception. Ethylene insensitivity is conferred by dominant mutations in the *ETR1* gene [44–48]. It has also been reported that the *Lotus japonicus* line transformed with the *Arabidopsis thaliana ETR1–1* gene delayed petal senescence and showed ethylene insensitivity [49]. However, unlike the mutant line produced by exogenous DNA introduction, the phenotype of *C416* was caused by a mutation in the endogenous *ETR1* gene. At the transcriptome level, from S1 to S2, EBF1, which negatively regulates the ethylene signaling pathway by mediating the degradation of EIN3/EIL [50, 51], was significantly upregulated in *C416*, whereas no obvious change was detected in the WT; EIN3, which is a positive regulator of ethylene response, showed no significant change in *C416*, but was upregulated in the WT. In *C416*-S2, ERF transcription factors, including *WIN1*, *ERF010*, *PAP2–3*, and *TINY*, exhibited lower expression levels compared to those in WT-S2. Three DEGs (CUFF.55905, CUFF.8415, and CUFF.8416) involved in nodulation were detected by RNA-seq in WT vs. *C416*, all of which were upregulated in *C416*. As ethylene has been demonstrated to play a special role in regulating symbiotic nitrogen-fixation and nodulation organogenesis in legumes [18–20], *C416* might also be a useful tool to study the effect of ethylene on nodulation by using mutants of endogenous genes in *Lotus japonicus*, rather than mutant lines through introduction of exogenous genes by transgenic techniques.

Petals in flowering plants senesce after pollination or according to the time following flower opening, regardless of whether they are pollinated or not. In some species, pollination dramatically shortens floral life span, as seen in orchid flowers, petunias, tobacco and carnations, senescence is triggered by ethylene following contact between pollen and the stigma surface [52–58]. When considering the different developmental stages, the number of DEGs in S1 vs. S2 was much higher than that in S2 vs. S3. Meanwhile, WT-S2 vs. *C416*-S2 had more DEGs than those of WT-S1 vs. *C416*-S1 (Fig. 5B). These results indicate that pollination-related processes might stimulate flower senescence in *Lotus japonicus*.

Flower senescence is delayed but not completely suppressed in *C416*, indicating that besides ethylene, other phytohormones might be also involved in the regulation of flower senescence in *C416*. Generally speaking, focused on flower senescence, ethylene and ABA promote senescence, whereas cytokinins and GA inhibit this process; auxin exhibits dual effects that can both accelerate or delay plant senescence. Compared to the above hormones, SA, JA and BR were also reported to influence petal senescence, however, the effects of SA, JA and BR on petal senescence are largely undefined [21, 59–62].

GA-insensitive dwarf 2 protein (GID2) is essential for GA-mediated DELLA protein degradation [63]. Here, GID2 was significantly upregulated from WT-S1 to WT-S2, whereas no obvious change was detected in *C416*-S1 to *C416*-S2. Endogenous cytokinin levels decline during the leaf aging process, and exogenous application can delay leaf and petal senescence [64, 65]. In *C416*-S2, *ARR11* (Arabidopsis response regulator 11), which is involved in the cytokinin-activated signaling pathway, was upregulated compared to that in WT-S2; ARR9 and ARR 12 were significantly upregulated from *C416*-S1 to *C416*-S2, whereas no obvious change was detected in WT-S1 vs. WT-S2. AHP4, which was inferred to play a negative role in several cytokinin responses [66] exhibited no significant changes in *C416*-S1 vs. *C416*-S2 when it was upregulated from WT-S1 to WT-S2.

SA is considered as promoter of leaf senescence, however, it has been found that exogenous application of SA increased the vase life of lisianthus, gladiolus and chrysanthemums [67–69]. In addition, RNA sequencing and microarray analysis showed that SA biosynthesis related factors were downregulated in opening stage while upregulated in senescent stage [70]. The SA response factor TGA3 exhibited no obvious change from *C416*-S1 to *C416*-S2, whereas it was significantly upregulated in WT; furthermore, its expression level in *C416*-S2 was lower than that in WT-S2. Exogenous methyl jasmonate application accelerated petal senescence in *Rosa hybrida*, *Petunia hybrid*, and *Dendrobium* spp. by increasing ACC levels to elevate ethylene production [71, 72]. However, JA treatment delayed tepal senescence of Iris flowers by 2 days [73]. In *C416*-S2, *JAR1*, which encodes jasmonic acid amido synthetase, was downregulated compared to that in the WT-S2. MYC2, which positively regulates a series of JA-dependent responses [74], was significantly downregulated from *C416*-S1 to *C416*-S2, whereas no obvious changes were observed in WT-S1 vs. WT-S2. In Arabidopsis, BR receptor mutant bri1 shows a premature leaf senescence phenotype [75]. Exogenous BR delayed senescence in *Carica papaya* L. leaves [62]. During rose petal senescence, the accumulation of putative BR SIGNALING KINASE (BSK) proteins were increased [76]. Here, the brassinosteroid insensitive 1-associated receptor kinase 1 (BAK1) was significantly upregulated from *C416*-S1 to *C416*-S2, whereas no obvious change was detected in WT-S1 vs. WT-S2.

Auxin exhibits dual effects that can accelerate or delay plant senescence. Several studies have demonstrated that auxin functions as a negative regulator of senescence, as exogenous application to the detached leaves of *Arabidopsis thaliana* resulted in the downregulation of *SAG12* [77]. The *ORESARA14* (encoding ARF2) knockout mutant line, as well as the *YUCCA6* (auxin biosynthetic gene) overexpression line showed delayed leaf senescence in *Arabidopsis thaliana* [78, 79]. In contrast, auxin can also promote leaf senescence by upregulating *SAUR*, which is a positive regulator of the senescence process [80, 81]; GH3 was upregulated during dark-induced senescence in *Arabidopsis thaliana* [78]. In the present study, SAUR-like auxin responsive protein and GH3.6 were significantly upregulated from WT-S1 to WT-S2, whereas no obvious changes were detected in *C416*-S1 vs. *C416*-S2. ARF, IAA13, and IAA19 were significantly upregulated from *C416*-S1 to *C416*-S2, and no obvious changes were detected in WT-S1 vs. WT-S2. Meanwhile, *ARF1*, *ARG7*, *X10A* and auxin-induced protein encoding gene *15A* involved in the auxin metabolic pathway were upregulated in *C416*-S2 compared to those in the WT-S2. This indicated that auxin also had a dual effect on flower senescence in *Lotus japonicus*.

ABA acts as a positive regulator of senescence. In ethylene-insensitive plants, ABA has been reported to play a predominant roles in flower senescence [82]. PP2C is a negative regulator of abscisic acid signaling by inhibiting the activity of SnRK2 and negatively regulates *Arabidopsis* leaf senescence [83]. There were no significant changes in *C416*-S1 vs. *C416*-S2, when significantly upregulated from WT-S1 to WT-S2. In *C416*-S2, the serine/threonine-protein kinase *SRK2I*, which is a core factor of the ABA-activated signaling pathway, was upregulated compared to that in WT-S2. Thus, ABA might be activated to promote flower senescence when the regulatory effects of ethylene are defective in *C416*.

## Conclusions

Mutation breeding by heavy-ion beam irradiation has the potential to improve flower longevity in legume plants. *C416*, which displayed delayed flower senescence, was induced by carbon-ion beam irradiation. This study aimed to identify the candidate genes responsible for the delayed senescence of *C416*. By association analysis of multi-omics and genetic mapping, CUFF.40834, which encodes *ETR1*, was identified as the most probable candidate. *C416* is insensitive to exogenous ACC application. This is the first reported mutant caused by endogenous ethylene-related genes in *Lotus japonicus*, rather than through the introduction of exogenous genes by transgenic techniques. Based on the present results and discussion, we provided a schematic of expression profile of plant hormone signaling transduction genes in flower senescence delayed mutant *C416* (Fig. 7). In brief, the ethylene, JA, and SA signaling pathways were negatively regulated in *C416*, whereas the BR and cytokinin signaling pathways were positively regulated, and auxin exhibited dual effects on flower senescence in *Lotus japonicus*. The ABA signaling pathway was positively regulated in *C416*, and ABA might be the major phytohormone factor that promotes senescence when the ethylene signaling pathway is blocked in *Lotus japonicus*. This study is informative for elucidation of the molecular mechanism of delayed flower senescence in *C416*, lays a foundation for candidate flower senescence gene identification in *Lotus japonicus*, and provides another perspective for the improvement of flower longevity in legume plants by heavy-ion beam.

## Methods

### Carbon-ion beam irradiation and plant growth conditions

The seeds of wild-type (WT) *Lotus japonicus* (ecotype: Miyakojima MG-20 and Gifu B-129) were bought from Frontier Science Research Center, University of Miyazaki, Japan. The dry seeds of laboratory wild-type (WT) *Lotus japonicus* (ecotype: Miyakojima MG-20) were exposed to ^12^C^6+^ ions at 400 Gy (energy, 80.55 MeV/nucleon; average LET within samples, 31 keV/μm) generated by the HIRFL at the Institute of Modern Physics, Chinese Academy of Sciences. The flower senescence-delayed mutant line *C416* of *Lotus japonicus* (background Miyakojima MG-20 ecotype) was isolated from the M_2_ population. Mutation screening methods have been described in our previous study [35]. Plants were grown under greenhouse conditions: 22 °C with a photoperiod of 18 h-light/6 h-dark, with a light intensity of 6000 lx.

### Whole genome re-sequencing and mutation verification

Young leaves of the WT line and six M_4_ lines (C16, yellow leaves; *C416*, delayed flower senescence; C83, C221, C328, and C434, inconspicuous phenotypes) derived from carbon-ion beam were used for genomic DNA extraction by cetyltrimethylammonium bromide (CTAB) method. The extracted DNA was sequenced using IIIumina HiSeqTM 2500 system (Illumina, Inc.; San Diego, CA, USA) at the Biomarker Technologies Company (Beijing, China). Bioinformatics analysis was performed as previously described [31, 41]. In short, the clean data were mapped to the reference genome of Lotus_r3.0 (ftp://ftp.kazusa.or.jp/pub/lotus/lotus_r3.0/) by using the Burrows-Wheeler Alignment v.0.7.15 tool [84] and SAMtools v.1.3.1 [85]. Qualimap v.2.2.1 was used to examine the sequencing alignment data, and the overall view of the data is shown in supplementary Table S2. Variant calling was carried out using VarScan 2 (v.3.9) algorithms [86]. The variants that were shared between two or among more sequenced lines were ruled out because they may preexist in the primordial WT lines. The variants with allele frequencies above 0.75 were called homozygous mutations, whereas all others were called heterozygous. Here, we only considered homozygous mutations in *C416* genome as it is a stable mutant. After verified by IGV, the remaining variants were annotated using the SnpEff toolbox to evaluate the effects of mutations on gene functions, based on the gene structure predictions in the annotations of Lotus_r3.0 (ftp://ftp.kazusa.or.jp/pub/lotus/lotus_r3.0/Lj3.0_gene_models.gff3.gz). Meanwhile, several randomly selected variants were sequenced by sanger sequencing. Primers were designed using Primer3 (ver. 0.4.0; http://bioinfo.ut.ee/primer3-0.4.0/), and the sequences of all primers are shown in supplementary Table S3.

### Genetic rough mapping

*C416* plants were crossed with another *Lotus japonicus* wild type (WT) Gifu B-129. DNA was isolated from F_2_ plants that displayed homozygous recessive phenotypes using the CTAB method. Several simple sequence repeat (SSR) markers (http://www.kazusa.or.jp/lotus/markerdb_index.html) were selected to determine the approximate chromosomal position of the mutation through polyacrylamide gel electrophoresis. Primer information for these markers is shown in supplementary Table S4.

### RNA isolation and sequencing

For the flower senescence-delayed mutant line *C416*, petals were collected during three periods: 2 days before pollination (Stage 1, S1), 2 days after pollination (Stage 2, S2) and 8 days after pollination (Stage 3, S3). WT group included only the first two periods (S1 and S2) as the petals had already faded in S3. Each group contained at least three biological replicates. Total RNA was extracted using the RNAprep Pure Plant Kit according to the manufacturer’s protocol (TIANGEN, China). RNA quality was assessed using NanoPhotometer spectrophotometer (IMPLEN, CA, USA), Qubit RNA Assay Kit in Qubit 2.0 Fluorometer (Life Technologies, CA, USA), and RNA Nano 6000 Assay Kit of the Agilent Bioanalyzer 2100 system (Agilent Technologies, CA, USA). Samples were sent to the Biomarker Technologies Company (Beijing, China) for library preparation and sequencing on an Illumina Hiseq 4000 platform. Adapter sequences and low-quality sequence reads were removed from the datasets. Clean reads were mapped to the reference genome sequence (Table S5). Differential expression analysis of two conditions/groups was performed using the DESeq R package. Differential gene expression was determined by the standard of FC ≥ 2, with a false discovery rate of *p* ≤ 0.01. Eighteen genes were selected randomly to validate the expression profiles of RNA-seq by real-time quantitative reverse transcription PCR (RT-qPCR), for which the primer information is shown in supplementary Table S6.

### Real-time quantitative reverse transcription PCR

Primers were designed using the Primer 3 program (http://primer3.ut.ee/#userconsent#), and synthesized by Sangon Biotech (China). cDNA was synthesized using the Transcriptor First Strand cDNA Synthesis Kit (Roche, Germany). RT-qPCR was performed using Rotor-Gene Q (Qiagen, Germany) following the FastStart Essential DNA Green Master kit (Roche, Germany). The PCR cycles consisted of initial denaturation at 95 °C for 10 min, followed by 40 cycles of 95 °C for 15 s and 60 °C for 60 s. The expression level of β-actin (Lj2g3v0916200, CUFF.35861.2) was used as an endogenous control for normalization.

### Bioinformatic analysis of protein

Based on the amino acid sequence of target protein, online systems including SWISS-MODEL (https://swissmodel.expasy.org/), Plant-mPLoc (http://www.csbio.sjtu.edu.cn/bioinf/plant-multi/), PredictProtein (https://predictprotein.org/), and Expasy ProtScale (https://web.expasy.org/cgi-bin/protscale/protscale.pl/) were used to perform a protein 3D structure modeling, subcellular localization prediction, and physicochemical property analysis [87–89]. The amino acid sequence of target protein was also submitted to Basic Local Alignment Search Tool (BLAST) for sequence similarity calculating and phylogenetic analysis.

### Response to exogenous ACC treatment

For the triple response assay, dry seeds were pretreated with abrasive paper, surface-sterilized with bleach for 15 min, washed with sterile water five times, and then sown on 1/2 Murashige and Skoog (MS) medium plates containing 0, 2, 5, 10, and 50 μM ACC. The medium plates were wrapped with aluminum foil, transferred to the growth room, and cultured in a vertical direction. The image of the seedlings was captured at 10th day after the transfer. The length of the hypocotyls was analyzed using ImageJ software. For the gene expression model assay involved in ethylene synthesis or signal transduction, seeds were sown on 1/2 MS medium plates for 2 days, then transferred to a medium containing 5 μM ACC and harvested at 0, 2, 4, and 6 h after transfer. Detached flowers of different developmental stages were floated in liquid containing 0 and 100 μM ACC, and the image was captured after 7 days.

### Statistical analysis

Statistical significance of the results was examined using one-way ANOVA with a multiple comparison test, t-test or chi-squared test using SPSS statistics 17.0. Differences between samples were considered significant when the *p*-value was less than 0.05.

## Supplementary Information

**Additional file 1: Fig. S1.** The amplification pattern of genetic mapping markers. “P” indicates the band pattern of the MG20 ecotype genomic DNA, “G” indicates the band pattern of the Gifu B-129 ecotype genomic DNA. 1–23 represent the individual plant that carrying homozygous recessive gene in the F_2_ population.

**Additional file 2: Fig. S2.** Verification of a part of detected mutations by Sanger sequencing.

**Additional file 3: Fig. S3.** Verification of DNA sequence variation of candidate gene CUFF.40834.

**Additional file 4: Fig. S4.** Fold changes of 18 DEGs in RNA-Seq.

**Additional file 5: Fig. S5.** Relative expressions of 18 DEGs tested by RT-qPCR.

**Additional file 6: Table S1.** Homozygous variants detected in *C416*.

**Additional file 7: Table S2.** Whole genome re-sequencing data generated and mapping to the Lotus japonicus genome.

**Additional file 8: Table S3.** List of primers used for sanger sequencing.

**Additional file 9: Table S4.** List of primers used for genetic mapping.

**Additional file 10: Table S5.** RNA-sequencing data generated and mapping to the *Lotus japonicus* genome.

**Additional file 11: Table S6.** List of primers used for RT-qPCR.

## Data Availability

Data of RNA-seq in this study is available in NCBI with accession number SRP115872. The whole-genome sequencing data in this study have been deposited in the Genome Sequence Archive (Genomics, Proteomics & Bioinformatics 2017) in in National Genomics Data Center (Nucleic Acids Res 2020), Beijing Institute of Genomics (China National Center for Bioinformation), Chinese Academy of Sciences, under accession number CRA002796 that are publicly accessible at https://bigd.big.ac.cn/gsa.

## Abbreviations

ACC: 1-Aminocyclopropane 1-carboxylic acid
ABA: Abscisic acid
BR: Brassinosteroid
CTAB: Cetyltrimethylammonium bromide
DEGs: Differential expression genes
DSBs: DNA double stranded breaks
HIRFL: Heavy Ion Research Facility In Lanzhou
InDels: Insertions and deletions
JA: Jasmonic acid
LET: Linear energy transfer
MS: Murashige and Skoog
NGS: Next generation sequencing
RT-qPCR: Real-Time Quantitative reverse transcription Polymerase Chain Reaction
RBE: Relative biological effectiveness
SA: Salicylic acid
SBSs: Single base substitutions
WT: Wild type
