# Supplementary material for: Genome and transcriptome-based characterization of high energy carbon-ion beam irradiation induced delayed flower senescence mutant in Lotus japonicus
Source: BMC Plant Biol. 2021 Nov 3;21:510. doi: 10.1186/s12870-021-03283-0 (PMC8564971; doi:10.1186/s12870-021-03283-0)
Supplement: Supplementary file 1 — Additional file 1: Fig. S1. The amplification pattern of genetic mapping markers. “P” indicates the band pattern of the MG20 ecotype genomic DNA, “G” indicates the band pattern of the Gifu B-129 ecotype genomic DNA. 1–23 represent the individual plant that carrying homozygous recessive gene in the F2 population. [file 12870_2021_3283_MOESM1_ESM.docx]

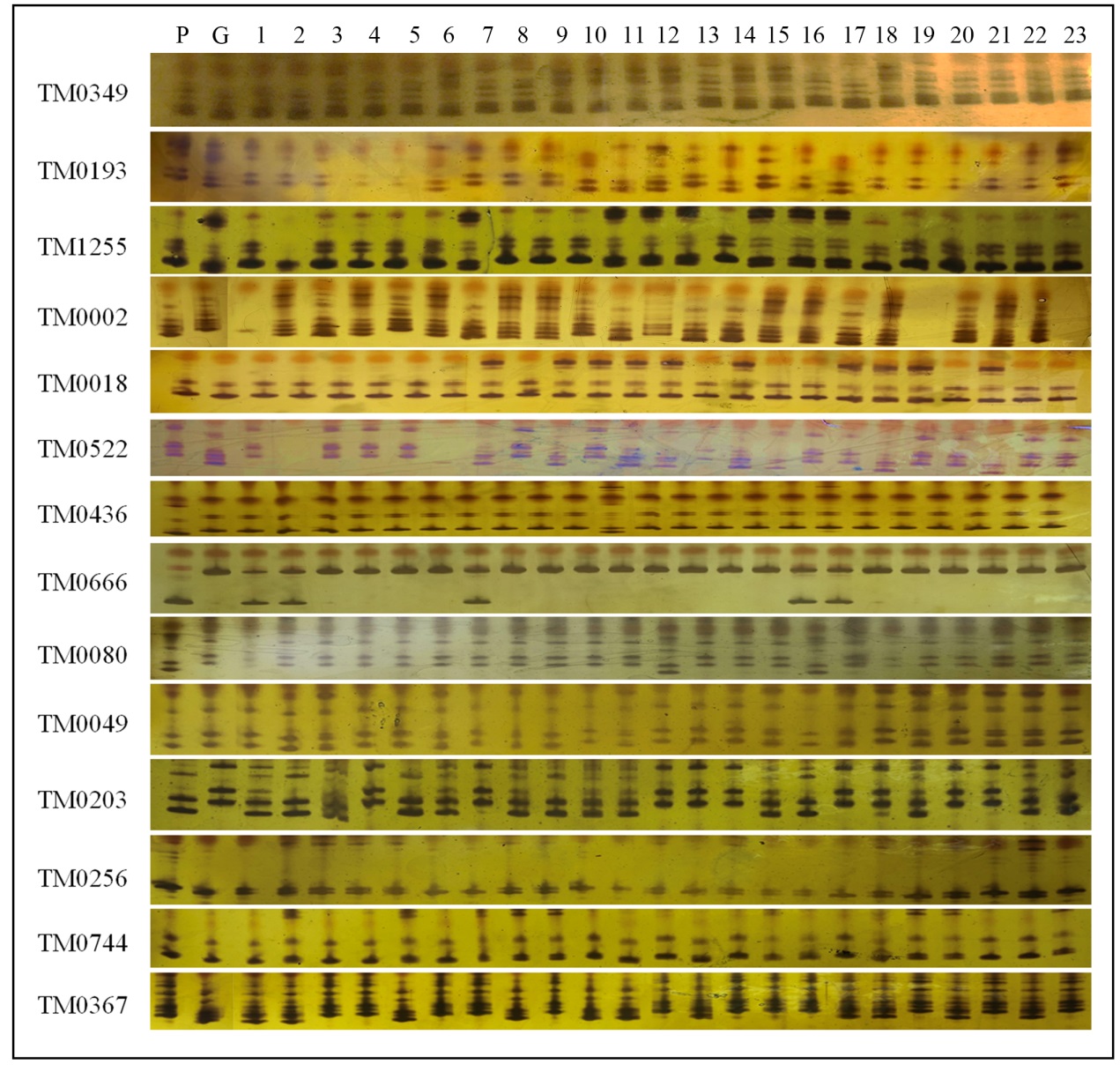


**Fig. S1.** The amplification pattern of genetic mapping markers. “P” indicates the band pattern of the MG20 ecotype genomic DNA, “G” indicates the band pattern of the Gifu B-129 ecotype genomic DNA. 1–23 represent the individual plant that carrying homozygous recessive gene in the F_2_ population.
