# Supplementary figures and images for: Genome and transcriptome-based characterization of high energy carbon-ion beam irradiation induced delayed flower senescence mutant in Lotus japonicus
Source: BMC Plant Biol. 2021 Nov 3;21:510. doi: 10.1186/s12870-021-03283-0 (PMC8564971; doi:10.1186/s12870-021-03283-0)

**
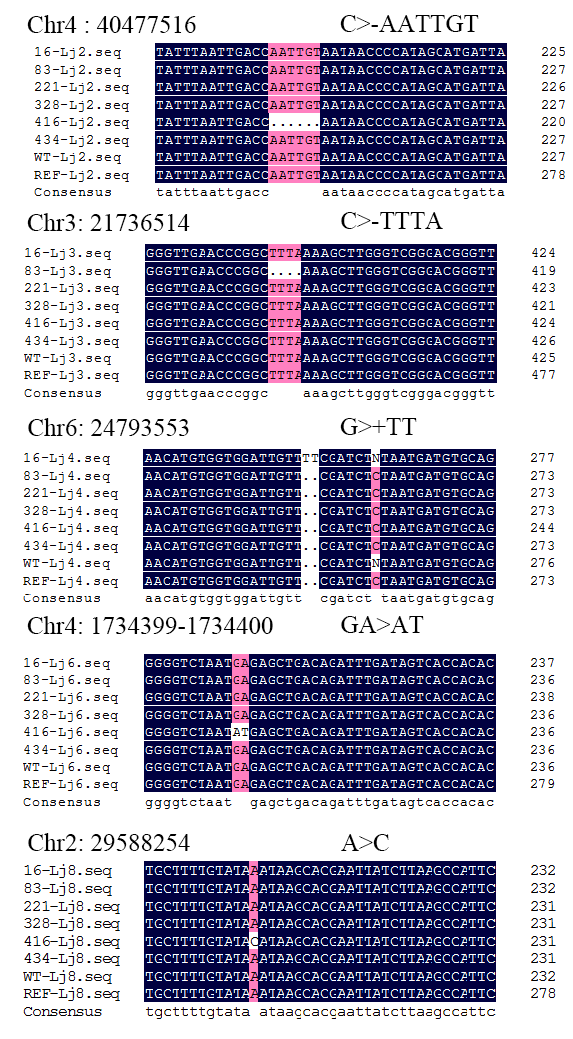
**

**Fig. S2.** Verification of a part of detected mutations by Sanger sequencing.

Supplement: Supplementary file 2 — Additional file 2: Fig. S2. Verification of a part of detected mutations by Sanger sequencing. [file 12870_2021_3283_MOESM2_ESM.docx]

**
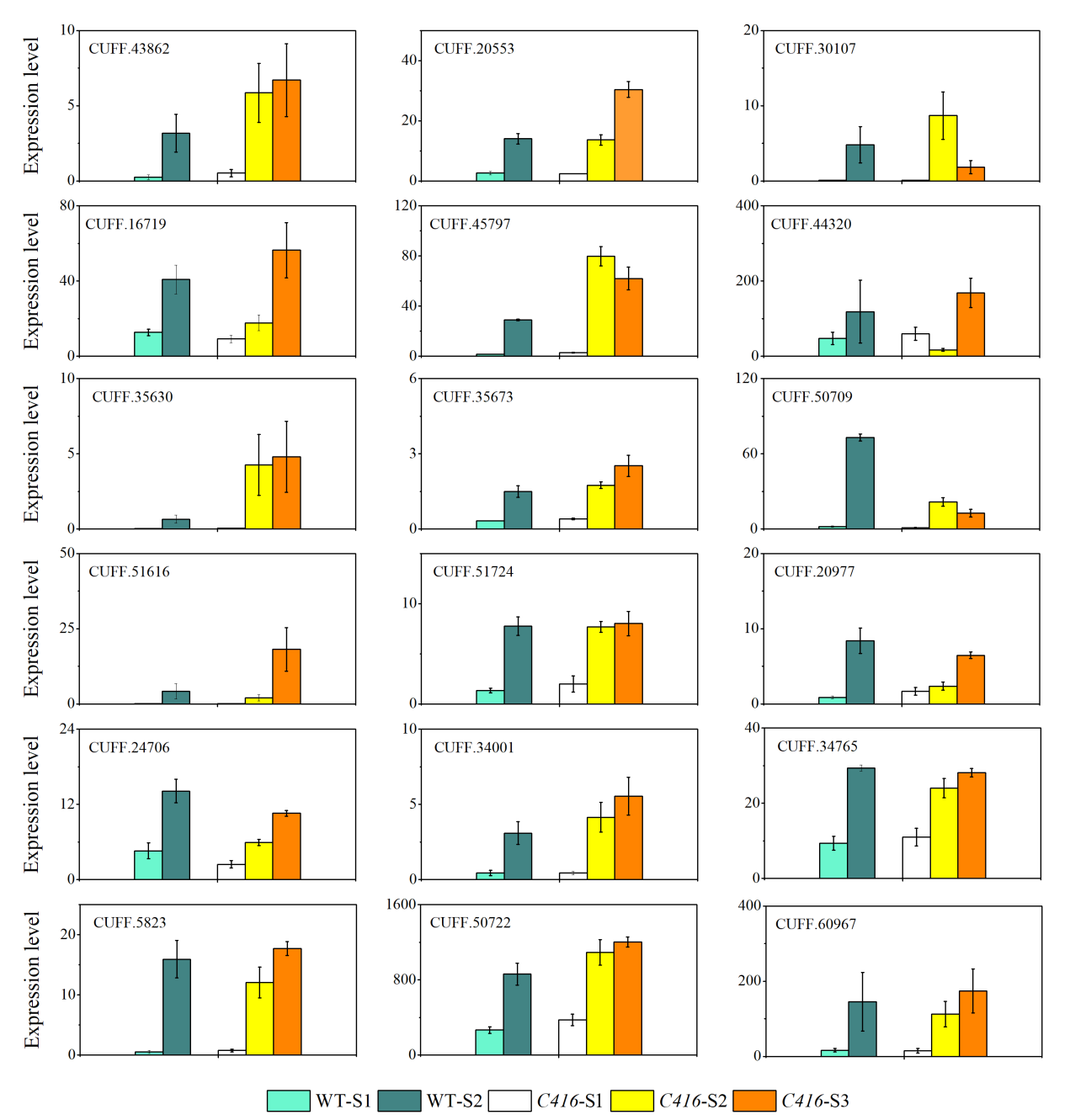
**

**Fig. S4.** Fold changes of 18 DEGs in RNA-Seq.

Supplement: Supplementary file 4 — Additional file 4: Fig. S4. Fold changes of 18 DEGs in RNA-Seq. [file 12870_2021_3283_MOESM4_ESM.docx]

**
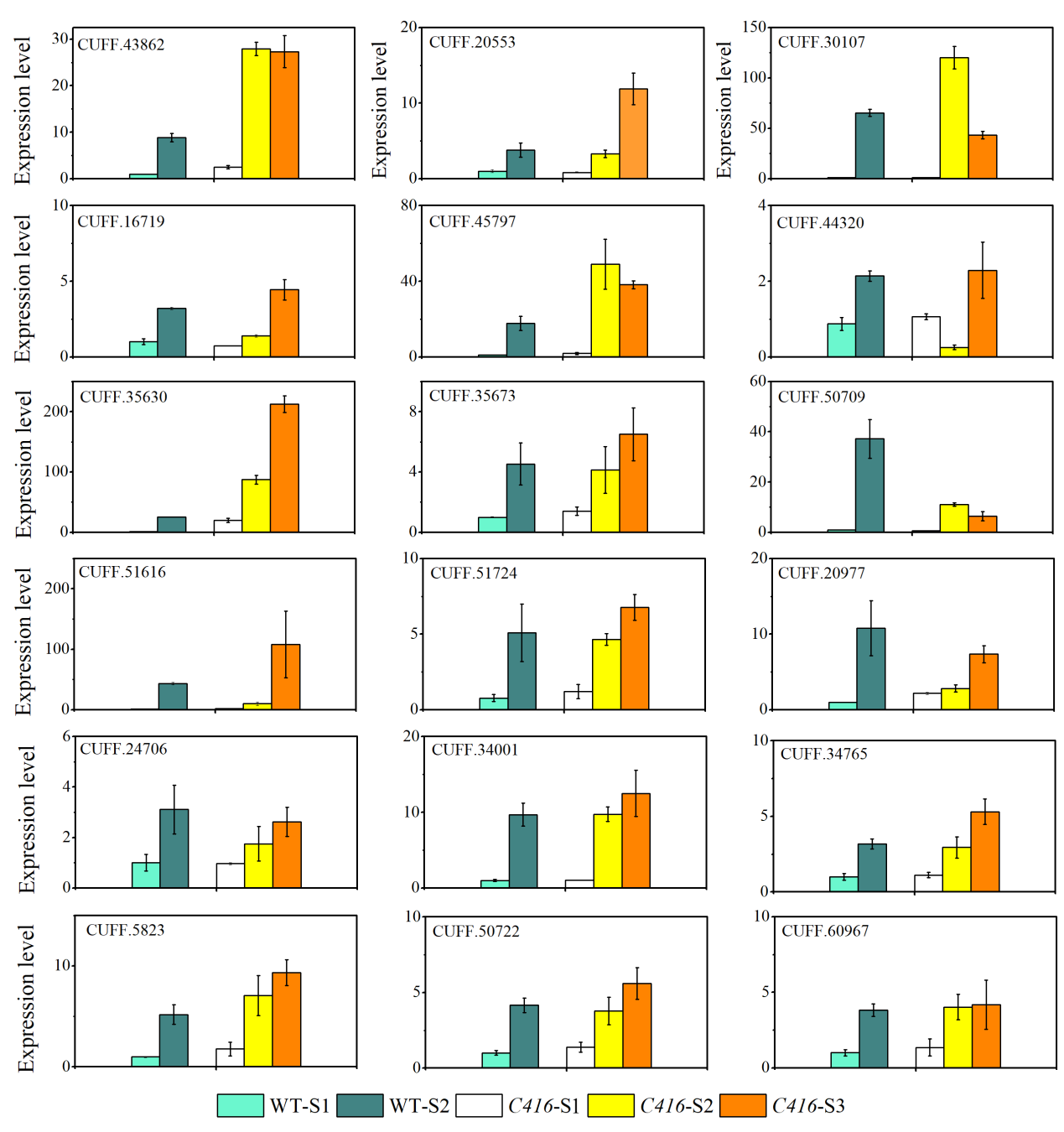
**

**Fig. S5.** Relative expressions of 18 DEGs tested by RT-qPCR.

Supplement: Supplementary file 5 — Additional file 5: Fig. S5. Relative expressions of 18 DEGs tested by RT-qPCR. [file 12870_2021_3283_MOESM5_ESM.docx]
