# Supplementary material for: Genome and transcriptome-based characterization of high energy carbon-ion beam irradiation induced delayed flower senescence mutant in Lotus japonicus
Source: BMC Plant Biol. 2021 Nov 3;21:510. doi: 10.1186/s12870-021-03283-0 (PMC8564971; doi:10.1186/s12870-021-03283-0)
Supplement: Supplementary file 3 — Additional file 3: Fig. S3. Verification of DNA sequence variation of candidate gene CUFF.40834. [file 12870_2021_3283_MOESM3_ESM.docx]

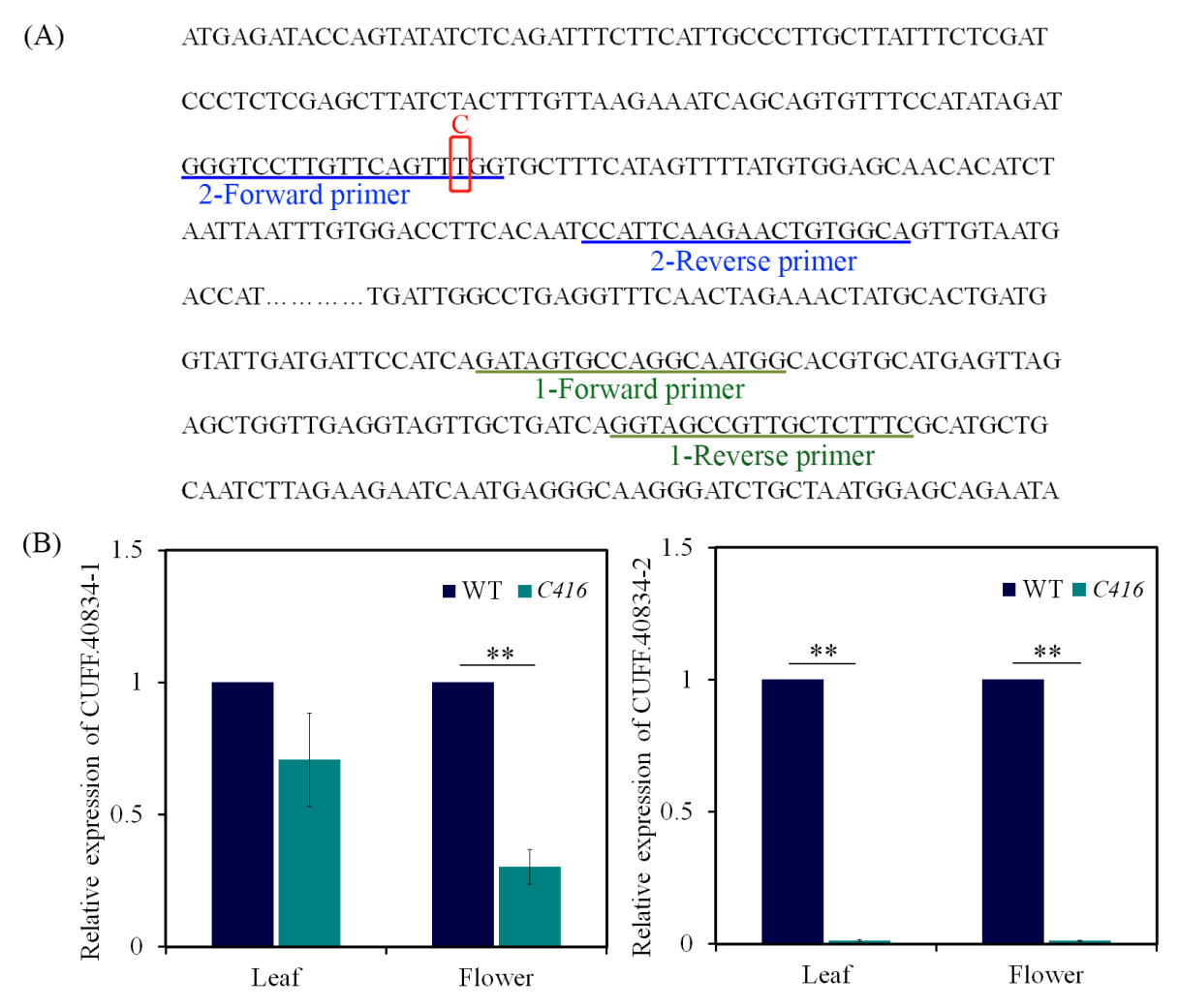


**Fig. S3.** Verification of DNA sequence variation of candidate gene CUFF.40834. (A) Sequence of CUFF.40834 and the position of primers for RT-qPCR that designed to assay the expression level of CUFF.40834. The red frame and letter showed the mutation detected by re-sequencing. The underline sequences indicated the position of primers for RT-qPCR. (B) Relative expression of CUFF.40834 in C416 compared to that of WT using the above two pairs of primers. The data points are mean ± standard error of three replications, asterisks indicate significant difference between WT and C416 by Student’s t-test (** p < 0.01).
