# Supplementary material for: Genome and transcriptome-based characterization of high energy carbon-ion beam irradiation induced delayed flower senescence mutant in Lotus japonicus
Source: BMC Plant Biol. 2021 Nov 3;21:510. doi: 10.1186/s12870-021-03283-0 (PMC8564971; doi:10.1186/s12870-021-03283-0)
Supplement: Supplementary file 6 — Additional file 6: Table S1. Homozygous variants detected in C416. [file 12870_2021_3283_MOESM6_ESM.docx]

**Table S1** Homozygous variants detected in *C416.*

| Chromosome | Position | Reference | Alteration | Type | Mutation region | Gene ID |
| --- | --- | --- | --- | --- | --- | --- |
| Lj3.0_chr0 | 89482722 | T | -G | Del | downstream | Lj0g3v0184129 |
| Lj3.0_chr0 | 149463384 | G | -CATAACAACAGAAAA | Del | exon | Lj0g3v0287639 |
| Lj3.0_chr0 | 160061476 | T | -TGGTACA | Del | downstream | Lj0g3v0306699 |
| Lj3.0_chr0 | 42214441 | G | +TTTCTCAA | Ins | upstream | Lj0g3v0099519 |
| Lj3.0_chr0 | 112185785 | G | -GGAATAGA | Del | upstream | Lj0g3v0219509 |
| Lj3.0_chr0 | 188502010 | G | A | SBS | upstream | Lj0g3v0358129 |
| Lj3.0_chr0 | 5211768 | T | A | SBS | upstream | Lj0g3v0013759 |
| Lj3.0_chr0 | 18170144 | A | T | SBS | exon | Lj0g3v0055949 |
| Lj3.0_chr0 | 48986237 | G | A | SBS | upstream | Lj0g3v0112469 |
| Lj3.0_chr0 | 76463183 | G | A | SBS | upstream | Lj0g3v0162189 |
| Lj3.0_chr0 | 85035683 | G | A | SBS | downstream | Lj0g3v0176049 |
| Lj3.0_chr0 | 119726295 | C | A | SBS | upstream | Lj0g3v0230969 |
| Lj3.0_chr0 | 125393533 | G | A | SBS | upstream | Lj0g3v0241629 |
| Lj3.0_chr0 | 146760633 | G | A | SBS | upstream | Lj0g3v0283149 |
| Lj3.0_chr0 | 157428073 | A | T | SBS | 5'-UTR | Lj0g3v0302609 |
| Lj3.0_chr0 | 174990604 | T | C | SBS | 3'-UTR | Lj0g3v0334309 |
| Lj3.0_chr0 | 179349895 | G | T | SBS | downstream | Lj0g3v0342159 |
| Lj3.0_chr0 | 116649426 | T | C | SBS | downstream | Lj0g3v0225959 |
| Lj3.0_chr0 | 132582450 | G | A | SBS | intergenic region | Lj0g3v0255269-Lj0g3v0255289 |
| Lj3.0_chr0 | 80329592 | A | T | SBS | intergenic region | Lj0g3v0168459-Lj0g3v0168469 |
| Lj3.0_chr0 | 127169179 | G | A | SBS | intergenic region | Lj0g3v0245369-Lj0g3v0245389 |
| Lj3.0_chr1 | 37409712 | T | -A | Del | downstream | Lj1g3v3328930 |
| Lj3.0_chr1 | 34735657 | A | -T | Del | upstream | Lj1g3v3041520 |
| Lj3.0_chr1 | 26916558 | A | -TGCCCTTATGTGTTG | Del | 5'-UTR | Lj1g3v2295190 |
| Lj3.0_chr1 | 17111128 | C | +T | Ins | downstream | Lj1g3v1387030 |
| Lj3.0_chr1 | 5151001 | A | G | SBS | upstream | Lj1g3v0415110 |
| Lj3.0_chr1 | 10530270 | G | T | SBS | upstream | Lj1g3v0796420 |
| Lj3.0_chr1 | 11171610 | T | A | SBS | upstream | Lj1g3v0874330 |
| Lj3.0_chr1 | 16223190 | G | A | SBS | upstream | Lj1g3v1357920 |
| Lj3.0_chr1 | 26354398 | T | C | SBS | upstream | Lj1g3v2205360 |
| Lj3.0_chr1 | 35970940 | T | C | SBS | downstream | Lj1g3v3171160 |
| Lj3.0_chr1 | 53430754 | G | A | SBS | upstream | Lj1g3v4528610 |
| Lj3.0_chr1 | 55759980 | T | G | SBS | upstream | Lj1g3v4693000 |
| Lj3.0_chr1 | 36556798 | G | A | SBS | exon | Lj1g3v3218140 |
| Lj3.0_chr1 | 22711812 | G | A | SBS | upstream | Lj1g3v1911810 |
| Lj3.0_chr1 | 13132641 | C | A | SBS | downstream | Lj1g3v1062530 |
| Lj3.0_chr1 | 47632939 | C | T | SBS | intron | Lj1g3v4026060 |
| Lj3.0_chr2 | 8141957 | C | T | SBS | upstream | Lj2g3v0604380 |
| Lj3.0_chr2 | 12330864 | C | T | SBS | upstream | Lj2g3v0777200 |
| Lj3.0_chr2 | 29588504 | A | C | SBS | upstream | Lj2g3v1988780 |
| Lj3.0_chr2 | 8479940 | C | T | SBS | upstream | Lj2g3v0621120 |
| Lj3.0_chr2 | 23460564 | T | A | SBS | 3'-UTR | Lj2g3v1468410 |
| Lj3.0_chr2 | 23460563 | C | T | SBS | 3'-UTR | Lj2g3v1468410 |
| Lj3.0_chr3 | 38052958 | T | +TTC | Ins | upstream | Lj3g3v3043330 |
| Lj3.0_chr3 | 12534429 | G | T | SBS | downstream | Lj3g3v0964270 |
| Lj3.0_chr3 | 4271744 | T | G | SBS | upstream | Lj3g3v0429250 |
| Lj3.0_chr3 | 4271749 | T | A | SBS | upstream | Lj3g3v0429250 |
| Lj3.0_chr3 | 4271764 | A | G | SBS | upstream | Lj3g3v0429250 |
| Lj3.0_chr3 | 4982946 | T | C | SBS | exon | Lj3g3v0461750 |
| Lj3.0_chr4 | 24782377 | T | -GCATAA | Del | upstream | Lj4g3v1721930 |
| Lj3.0_chr4 | 28903182 | T | -G | Del | upstream | Lj4g3v2118240 |
| Lj3.0_chr4 | 40477516 | C | -AATTGT | Del | downstream | Lj4g3v3017340 |
| Lj3.0_chr4 | 6444891 | G | C | SBS | upstream | Lj4g3v0450840 |
| Lj3.0_chr4 | 17415945 | A | G | SBS | upstream | Lj4g3v1154470 |
| Lj3.0_chr4 | 33268349 | C | T | SBS | upstream | Lj4g3v2400980 |
| Lj3.0_chr4 | 1734399 | G | A | SBS | intron | Lj4g3v0149170 |
| Lj3.0_chr4 | 1734400 | A | T | SBS | intron | Lj4g3v0149170 |
| Lj3.0_chr5 | 22590526 | T | A | SBS | downstream | Lj5g3v1536600 |
| Lj3.0_chr5 | 24715179 | T | C | SBS | upstream | Lj5g3v1699450 |
| Lj3.0_chr6 | 24717334 | G | A | SBS | 3'-UTR | Lj6g3v2116920 |
| Lj3.0_chr6 | 9319704 | G | A | SBS | exon | Lj6g3v0920480 |
| Lj3.0_chr6 | 6605003 | G | A | SBS | upstream | Lj6g3v0658950 |
