# Supplementary material for: Genome and transcriptome-based characterization of high energy carbon-ion beam irradiation induced delayed flower senescence mutant in Lotus japonicus
Source: BMC Plant Biol. 2021 Nov 3;21:510. doi: 10.1186/s12870-021-03283-0 (PMC8564971; doi:10.1186/s12870-021-03283-0)
Supplement: Supplementary file 7 — Additional file 7: Table S2. Whole genome re-sequencing data generated and mapping to the Lotus japonicus genome. [file 12870_2021_3283_MOESM7_ESM.docx]

**Table S2** Whole genome re-sequencing data generated and mapping to the *Lotus japonicus* genome.

| Sample | Total Reads | Clean Bases(G) | Mapped Reads (%) | Depth (×) | ≥Q30 (%) |
| --- | --- | --- | --- | --- | --- |
| WT | 42,443,972 | 12.68 | 90.19 | 28.34 | 90 |
| *C16* | 32,003,256 | 9.60 | 98.35 | 21.46 | 90.77 |
| *C83* | 35,409,721 | 10.62 | 98.48 | 23.74 | 90.13 |
| *C221* | 35,178,902 | 10.55 | 98.57 | 23.59 | 90.76 |
| *C328* | 35,192,131 | 10.56 | 98.53 | 23.6 | 90.75 |
| *C416* | 47,751,954 | 14.26 | 97.16 | 31.87 | 89.95 |
| *C434* | 34,471,545 | 10.34 | 98.58 | 23.11 | 91.01 |
