# Supplementary material for: Genome and transcriptome-based characterization of high energy carbon-ion beam irradiation induced delayed flower senescence mutant in Lotus japonicus
Source: BMC Plant Biol. 2021 Nov 3;21:510. doi: 10.1186/s12870-021-03283-0 (PMC8564971; doi:10.1186/s12870-021-03283-0)
Supplement: Supplementary file 8 — Additional file 8: Table S3. List of primers used for sanger sequencing. [file 12870_2021_3283_MOESM8_ESM.docx]

**Table S3** List of primers used for sanger sequencing.

| Primer | Forward primer (5’-3’) | Reverse primer (5’-3’) |
| --- | --- | --- |
| Lj2-F | TTGCACCTCCTAGCCCTAAA | TTGGTGTCTGTTAGGTGTCTACTTG |
| Lj3-F | GGTCTTGCACTGATCCAATTC | TGTGATTCGTGAGAGGGTGA |
| Lj4-F | CATTCAATTTTCAGGCACACTC | GGGTTTTCGAAGAAGGAAGC |
| Lj6-F | TTAATGGTTGCGGATTACGG | GTTTTCGGCTCTCCACCATA |
| Lj8-F | TGTAACTGGGTGACCGTTAAAA | TGCTTGGAGACGAAATGATG |
