# Supplementary material for: Genome and transcriptome-based characterization of high energy carbon-ion beam irradiation induced delayed flower senescence mutant in Lotus japonicus
Source: BMC Plant Biol. 2021 Nov 3;21:510. doi: 10.1186/s12870-021-03283-0 (PMC8564971; doi:10.1186/s12870-021-03283-0)
Supplement: Supplementary file 9 — Additional file 9: Table S4. List of primers used for genetic mapping. [file 12870_2021_3283_MOESM9_ESM.docx]

**Table S4** List of primers used for genetic mapping.

| Marker | Chromosome | Forward primer (5’-3’) | Reverse primer (5’-3’) |
| --- | --- | --- | --- |
| TM0349 | I | AAACGAGTAGAAGGGAGGTC | TTCTCACTTTCACGCCGTTC |
| TM1255 | I | CCCAAAACCGATTTCGATTC | ACCTGATTCAGCTTTTACGC |
| TM0002 | II | AGCGATCTACATTCAAGAG | AGCGTTCTCTCAGTGTTG |
| TM0018 | II | GTTTGAGCAAGTTAGAGGTG | CGGATAAGAAAGGTAGAAGAG |
| TM0522 | II | GAGTACTCTTTCTCCATGTTACC | CGGGGAATAAAGAGGTAATC |
| TM0282 | III | AACTTTATACTCCGCGCTTC | TTTCGTTGAAATCAATTCCG |
| TM0704 | III | ACTATAGCCTGTTGAGAGGG | TGCTCATGGATTAAGGGGAC |
| TM0436 | III | ATGTTGTCTGTGTGTCTGTG | AAATTGATTGAAAAGGGGTG |
| TM0666 | III | CACATACATAAATCGCCAAC | TGTCCATTTGGAAGAGTGGC |
| TM0080 | III | AACAAAATACTAAACTATAGCAAAG | CGTCCCACAACTCTCTTTAC |
| TM0203 | III | CTGTTTTGACGAATCGATAG | TACGAAGGTTTCTTCATTCC |
| TM0256 | IV | GAAATTCTTTCCATTCATTG | AGAGAGATAGGGTTGCTCAC |
| TM0744 | V | CCCAGTGTTACATTACAGAGC | TTGTTCTGAAAATCCAGGTC |
| TM0367 | VI | CTCCTCCACCATCTTTGACC | GTTCGAACCCTAATGATGAC |
