# Supplementary material for: Genome and transcriptome-based characterization of high energy carbon-ion beam irradiation induced delayed flower senescence mutant in Lotus japonicus
Source: BMC Plant Biol. 2021 Nov 3;21:510. doi: 10.1186/s12870-021-03283-0 (PMC8564971; doi:10.1186/s12870-021-03283-0)
Supplement: Supplementary file 10 — Additional file 10: Table S5. RNA-sequencing data generated and mapping to the Lotus japonicus genome. [file 12870_2021_3283_MOESM10_ESM.docx]

**Table S5** RNA-sequencing data generated and mapping to the *Lotus japonicus* genome.

| Replicate | Sample | Total Reads | Mapped Reads(%) | Uniq Mapped Reads(%) | Multiple Map Reads(%) | ≥Q30 (%) |
| --- | --- | --- | --- | --- | --- | --- |
| Replicate 1 | WT-S1-1 | 67,049,760 | 78.64 | 69.24 | 9.4 | 90.43 |
|  | WT-S2-1 | 51,279,102 | 78.29 | 68.64 | 9.24 | 94.4 |
|  | *C416*-S1-1 | 44,872,562 | 78.96 | 69.63 | 9.5 | 94.12 |
|  | *C416*-S2-1 | 60,431,276 | 78.71 | 68.1 | 9.65 | 94.01 |
|  | *C416*-S3-1 | 65,164,292 | 78.63 | 69.17 | 9.23 | 93.58 |
| Replicate 2 | WT-S1-2 | 87,275,806 | 79.08 | 69.84 | 9.83 | 89.58 |
|  | WT-S2-2 | 89,866,944 | 77.32 | 68.1 | 9.33 | 93.15 |
|  | *C416*-S1-2 | 44,492,592 | 79.83 | 69.86 | 9.97 | 93.52 |
|  | *C416*-S2-2 | 55,224,624 | 73.68 | 64.47 | 9.5 | 94.11 |
|  | *C416*-S3-2 | 67,730,648 | 75.33 | 65.19 | 10.61 | 94.19 |
| Replicate 3 | WT-S1-3 | 48,592,184 | 79.05 | 69.55 | 9.21 | 93.6 |
|  | WT-S2-3 | 58,788,550 | 76.75 | 66.93 | 9.11 | 93.97 |
|  | *C416*-S1-3 | 52,368,258 | 79.39 | 69.89 | 9.46 | 93.92 |
|  | *C416*-S2-3 | 58,836,486 | 70.68 | 61.57 | 10.14 | 93.59 |
|  | *C416*-S3-3 | 65,229,978 | 79.48 | 68.74 | 10.73 | 93.63 |
