# Supplementary material for: Genome and transcriptome-based characterization of high energy carbon-ion beam irradiation induced delayed flower senescence mutant in Lotus japonicus
Source: BMC Plant Biol. 2021 Nov 3;21:510. doi: 10.1186/s12870-021-03283-0 (PMC8564971; doi:10.1186/s12870-021-03283-0)
Supplement: Supplementary file 11 — Additional file 11: Table S6. List of primers used for RT-qPCR. [file 12870_2021_3283_MOESM11_ESM.docx]

**Table S6** List of primers used for RT-qPCR.

| Gene | Forward primer (5’-3’) | Reverse primer (5’-3’) |
| --- | --- | --- |
| CUFF.5823 | CCATATCACCAGCTATTACCTCCC | TCATCTTTGAGTTCACCCACAAGA |
| CUFF.34765 | GACAAAGATGGGAGGGGAAGATAA | ATTTCTGCAACATCAACAACCTCC |
| CUFF.60967 | GGCAATGGTTCCTGTCTTCTTAAC | ATCAGTGTTTCCCTCTTTCACCTT |
| CUFF.50722 | GGTGCTGAGGATGAATTGAAACAT | CACAAGTGTCACTCGTGTAAACTC |
| CUFF.20553 | TCTTCATCTCTCTGCCTCTCTAGT | TGGTGTTGAATTGAATGCTGACTC |
| CUFF.20977 | AGGACAGGGATTATTGTTGTTCCA | CACTTCCTCATCCTTATCCCTCTG |
| CUFF.24706 | TGCAGGTTTTGTGTCACTCTACTA | ATGAGCTCAGTCGAAGTAACCTTT |
| CUFF.30107 | CAACAGCCTTTAATCCTCTTTGCA | GAGTAAAGACCAAGTGCGATGATG |
| CUFF.34001 | AATATGCTTAAACTCCGCCACATG | AGAGCAACAGAGGATTACAGTACC |
| CUFF.35630 | TGAGGCTGAGAAAGAGGTACAAAA | TCAGATTCATCCAGCAAAACAACC |
| CUFF.35673 | CATGAGTGAAAACCTTGGTTTGGA | ACATTGAGGGTACTTGGCTACTTT |
| CUFF.43862 | GCCTGCTTGCTGAGATGAATTTAT | ACTTTCTGCAGCTTGTTGTGAATT |
| CUFF.50709 | ATAGATGAATACCCTAACCGTGGC | TTGGTGAGTGGAAGAAGAAGTGAA |
| CUFF.51616 | CTTCAGAGCCAATAGTTCATGCAG | GATGATGAGAAGCCATGGTTGATG |
| CUFF.51724 | CTTCTTCCACCTATCTCTCACCAG | TGAAGCTTGTTTGGGATTTGCTAG |
| CUFF.44320 | TGGTGCCAATGAGCTTCTAATAGT | TTACCAGCAGCTACTTGAAAATGC |
| CUFF.40834-1 | GATAGTGCCAGGCAATGG | GAAAGAGCAACGGCTACC |
| CUFF.40834-2 | GGGTCCTTGTTCAGTTTGG | TGCCACAGTTCTTGAATGG |
| CUFF.32775 | CAAGAAGTACCACAGCTTACC | CATAGCCAAGAGAACCATCC |
| CUFF.16719 | CGTCACCACCTATGAAGAA | CTCTCACCACCAGATGTC |
| CUFF.45797 | CTGTGGAGGTGCCGAAGG | CTTGGTTCAGCATCTCTTGGAATG |
| Actin | GCATTGTTGGTCGTCCTCGT | TGTGCCTCATCCCCAACATA |
